# Hybrid Metal–Organic Framework‐Derived Dual Active Site Catalysts Enabling Surface‐Enriched Cathode and Modified Zinc Anode for Long‐Life Zinc–Air Batteries

**DOI:** 10.1002/advs.77650

**Published:** 2026-09-11

**Authors:** Ramasamy Santhosh Kumar, Duraisami Kaviyarasu, Dilmurod Sayfiddinov, Murugavel Kathiresan, Pandian Mannu, Chung‐Li Dong, Arul Manuel Stephan, Dong Jin Yoo

**Affiliations:** ^1^ Department of Energy Storage/Conversion Engineering of Graduate School BK21 FOUR, Hydrogen and Fuel Cell Research Center Jeonbuk National University Jeollabuk‐do Republic of Korea; ^2^ P. C. Ray Department of Chemistry National Institute of Technology Puducherry Karaikal Puducherry India; ^3^ Research Center for X‐ray Science Department of Physics Tamkang University Tamsui Taiwan; ^4^ CSIR‐Central Electrochemical Research Institute Karaikudi India; ^5^ Department of Life Science Jeonbuk National University Jeollabuk‐do Republic of Korea

**Keywords:** bifunctional cathode catalysts, hybrid metal‐organic frameworks, nanoparticles, Zn anode modification, Zn‐air batteries

## Abstract

The surface modification of zinc anodes and the development of effective cathode dual‐functional oxygen electrocatalysts remain of paramount interest for enhancing the cycle life of rechargeable zinc‐air batteries. The present work provides a sustainable synthesis of CoNi‐based composites that can be used as cathode and anode side catalysts in Zn‐air batteries. CoNi‐metals with hybrid‐metal organic frameworks (Hybrid‐MOFs) and MOF‐derived composites from curcumin‐melamine (C‐M) and benzene‐1,3,5‐tricarboxylic acid (BTC) were created for cathode and anode composites using reflux and hydrothermal techniques. In this instance, the cathode composite can be referred to as CoNi‐N‐C after annealing, whereas the anode composite can be referred to as CoNi‐BTC without annealing. Thereafter, the formation of CoNi‐N‐C and CoNi‐BTC composites was confirmed by microscopic, X‐ray photoelectron, and X‐ray diffraction techniques. As a result, structurally tailored CoNi‐N‐C and CoNi‐BTC composites perform efficiently in electrochemical processes such as oxygen evolution/reduction reactions (OER/ORR) and energy conversion devices (Zn‐air battery). Primarily, the CoNi‐N‐C catalyst exhibits the lowest overpotential (E_i = 10_ = 1.48 V) and better half‐wave potential (E_1/2_ = 0.781 V) in OER/ORR reactions; secondary CoNi‐BTC composites coated on the Zn anode have the highest specific capacity (756.3 mA h g_zn_^−1^) and 1000‐cycle charge–discharge stability in Zn‐air batteries.

## Graphical abstract

This work demonstrates the implementation of MOF‐assisted anode and cathode materials for prolonged cycling of zinc‐air batteries with strong redox capability and structural stability.

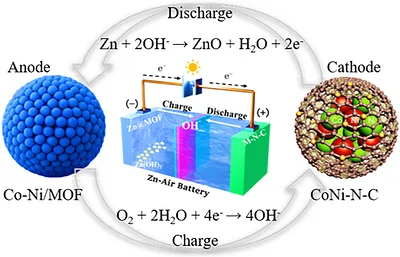

## 1 Introduction

Zinc‐air batteries (ZABs) have garnered a lot of interest due to their advantages, which include low cost, excellent safety, zero carbon emissions, good stability, and theoretical capacity that is based on zinc of 820 mA h kg^−1^ and energy density of 1086 W h kg^−1^ [1, 2, 3]. They have been regarded as the next‐generation power source in a number of domains, such as transportation, portable electronics, and distributed energy storage [4, 5]. Because they mutually require constant O_2_ and minimal Zn leaching as the reaction source, the air‐borne cathode and Zn anode within ZABs are essential components that help achieve high specific capacity and high round‐trip efficiency [6, 7, 8]. However, the primary obstacle to the practical use of ZABs is the slow kinetics at the air cathode caused by the high overpotential for the oxygen evolution and oxygen reduction reactions (OER/ORR) [9, 10]; Second, high‐surface‐area dendrites, uneven positioning of electrodes and electrodissolution, and continued corrosion that reduces electrolyte are all problems for zinc metal in an alkaline condition [8, 11]. Zn must be significantly modified or used in excess in order to combat this, which results in significant underutilization of its potential capacity [12, 13]. Alkaline electrolytes react with CO_2_ in the air to form insoluble carbonate salts on the cathode side, which physically blocks and chemically disables the porous air cathode in addition to consuming electrolyte irreversibly [4, 14, 15, 16]. In some superconcentrated electrolytes, very bidirectional Zn electrodeposition and dissolution of electrodes were also attained [12, 17].

Surface transformation, electrolyte alteration, and electrode structural 3D design are common techniques for enhancing the ability to reversibly and stably cycle Zn anodes throughout plating and stripping cycles [17, 18, 19]. Due to its remarkable outcomes and ease of application, surface alteration using metals, carbon compounds, polymer–based metal–organic frameworks, and oxide‐based coatings has drawn a lot of attention [20, 21, 22]. Metal‐organic frameworks (MOFs) are widely employed in a variety of applications, including sensors, catalysis, medicine dispersion, energy storage, and conversion. MOFs are created when metal ions and organic ligands coordinate [23, 24]. MOFs may therefore promote electrochemical processes on an electrode to attain high specific capacity by enhancing stability and rate efficiency and providing an abundance of redox‐active sites. This is because MOFs have an abundance of porous frameworks, extremely large surface areas, and an excess of adjustable sites [25, 26]. These results suggest the development of ZABs with excellent cycling stability, rate efficiency, and specific capacity by using substantial amounts of MOFs or nanomaterials produced from MOFs [17, 27]. Conventional alkaline electrolytes require the employment of bifunctional catalysts on the cathode side because the redox reaction of O_2_ takes place via a slow 4e^−^ O─O bond breakage and formation pathway [28, 29]. Nevertheless, the fundamental issue posed by a slow 4e^−^ route ORR mechanism at the cathode was not investigated. Developing dual‐functional catalysts for an air cathode or extending the life of the Zn anode through electrode engineering or electrolyte additives have been the main strategies utilized to increase the reversibility of ZABs [30, 31].

In the present work, a novel composite comprising CoNi nanoparticles embedded in a porous‐hybrid‐sphere structure (CoNi‐N‐C) was synthesized by introducing curcumin‐melamine (C‐M) and CoNi nanoparticles MOFs‐derived CoNi‐BTC as cathode‐anode electrodes in Zn‐air batteries. CoNi‐N‐C and CoNi‐BTC MOFs presented a microstructure in which the nanoparticles were embedded in the interconnected nitrogen‐doped carbon shell. Melamine acted as the source of carbon/nitrogen, and curcumin is a biomass chemical that is readily available as a source of carbon materials [29, 32]. Besides, BTC was not only beneficial for the formation of the microstructure of CoNi‐BTC, but it also acts as a barrier to prevent CoNi nanoparticles from escaping in an alkaline electrolyte during the Zn‐air battery anode electrode. The cathode electrode acts as hybrid‐MOFs‐derived CoNi‐N‐C, showing excellent ORR/OER (E_1/2_ = 0.781 V; E_i = 10_ = 1.48 V@10 mA) performances in alkaline solution due to the high specific surface area and abundant active sites, which facilitate efficient gas diffusion and electrolyte penetration. On the anode side, compared to Zn foil without MOF coating (724.9 mA h g_zn_^−1^; 500 cycles), CoNi‐BTC composites coated on the Zn anode had the highest specific capacity (756.3 mA h g_zn_^−1^) and 1000‐cycle charge–discharge stability with lower potential differences in Zn‐air batteries.

## 2 Experimental Section

### 2.1 Materials

Commercial curcumin, melamine (99.0%), cobalt (II) nitrate hexahydrate (97.7%), nickel (II) nitrate hexahydrate (99.99%) ammonium hydroxide solution (28.0%–30.0% NH_3_ basis), ethyl alcohol (95.0%), iridium (IV) oxide (99.9%), platinum on graphitized carbon (20 wt.% loading), potassium hydroxide (95.0%), and Benzene‐1,3,5‐tricarboxylic acid (BTC) (98% purity) were purchesed from Sigma‐Aldrich. Polyvinylpyrrolidone (PVP K‐30) and dimethylformamide (DMF) were sourced from Alfa Aesar and Daejung Chemicals. All chemicals were of analytical grade and used as received without further purification.

### 2.2 Synthesis of Hybrid‐Metal Organic Frameworks Derived CoNi‐N‐C Catalysts

Using a straightforward and cost‐effective reflux technique, bi‐metal nanoparticles were produced on a highly stable hybrid bio‐carbon surface. In brief, 150 mg of commercial curcumin and 50 mg of melamine were dissolved in 50 mL of ethanol with 50 mL of ammonium hydroxide solution and agitated for 1 h at room temperature. The mixture was stirred ultrasonically during dropwise addition of 5 mmol of cobalt nitrate and nickel nitrate, then left to stand for 10 min. The sonicated mixture was then stirred for 2 h at room temperature. Next, the solution was placed into a reflux system and stirred overnight at 130°C for 24 h. It was then centrifuged with a DI water/ethanol solution, and the obtained powder was pyrolyzed at 800°C for 2 h in an N_2_ atmosphere to produce the CoNi‐N‐C catalysts. The same procedure was followed for the synthesis of Co‐N‐C and Ni‐N‐C catalysts; these three catalysts were used as cathode catalysts in Zn‐air batteries.

In this synthesis, the amino groups of melamine and the phenolic compounds of curcumin are coupled, grafted, or create hydrogen bonds with the help of ammonium hydroxide, this first step synthesis process was called as, the San‐Yoo reaction product (full name; Santhosh Kumar‐Dong Jin Yoo reaction step). Finally, curcumin‐melamine (C‐M) nanocomposites were successfully formed, its was called hybrid metal organic frameworks (Hybrid‐MOFs).

### 2.3 Synthesis of Ni‐Co‐BTC MOFs

The bimetallic Ni‐Co‐BTC metalorganic framework was synthesized using a significantly altered process. The reaction scheme in Figure 5a depicts the synthesis process. In summary, 50 mL of a 1:1:1 DMF: ethanol: water solvent mixture was used to dissolve 0.5 mmol of cobalt nitrate, 2.5 mmol of nickel nitrate, 1.5 mmol of BTC, and 3.0 mmol of PVP. The mixture was moved to a 100 mL Teflon‐coated autoclave and heated to 150°C for 12 h after being stirred for 30 min at ambient temperature. PVP acted as a stabilizer, which was essential for spherical framework construction. After cooling to ambient temperature, the reaction solution was centrifuged for 10 min at 9000 rpm. To get rid of contaminants and unreacted starting materials, the residue was cleaned five times using ethanol and water. Grayish purple NiCo‐BTC MOFs were produced after the product was vacuum‐dried for 12 h at 60°C. Detailed descriptions of the materials, electrochemical characterizations, electrode manufacturing, and electrochemical calculations were given in the supporting materials file.

## 3 Results and Discussion

### 3.1 Synthesis and Characterization of CoNi‐N‐C, Co‐N‐C, and Ni‐N‐C as a Cathode Catalysts

**FIGURE 1:**
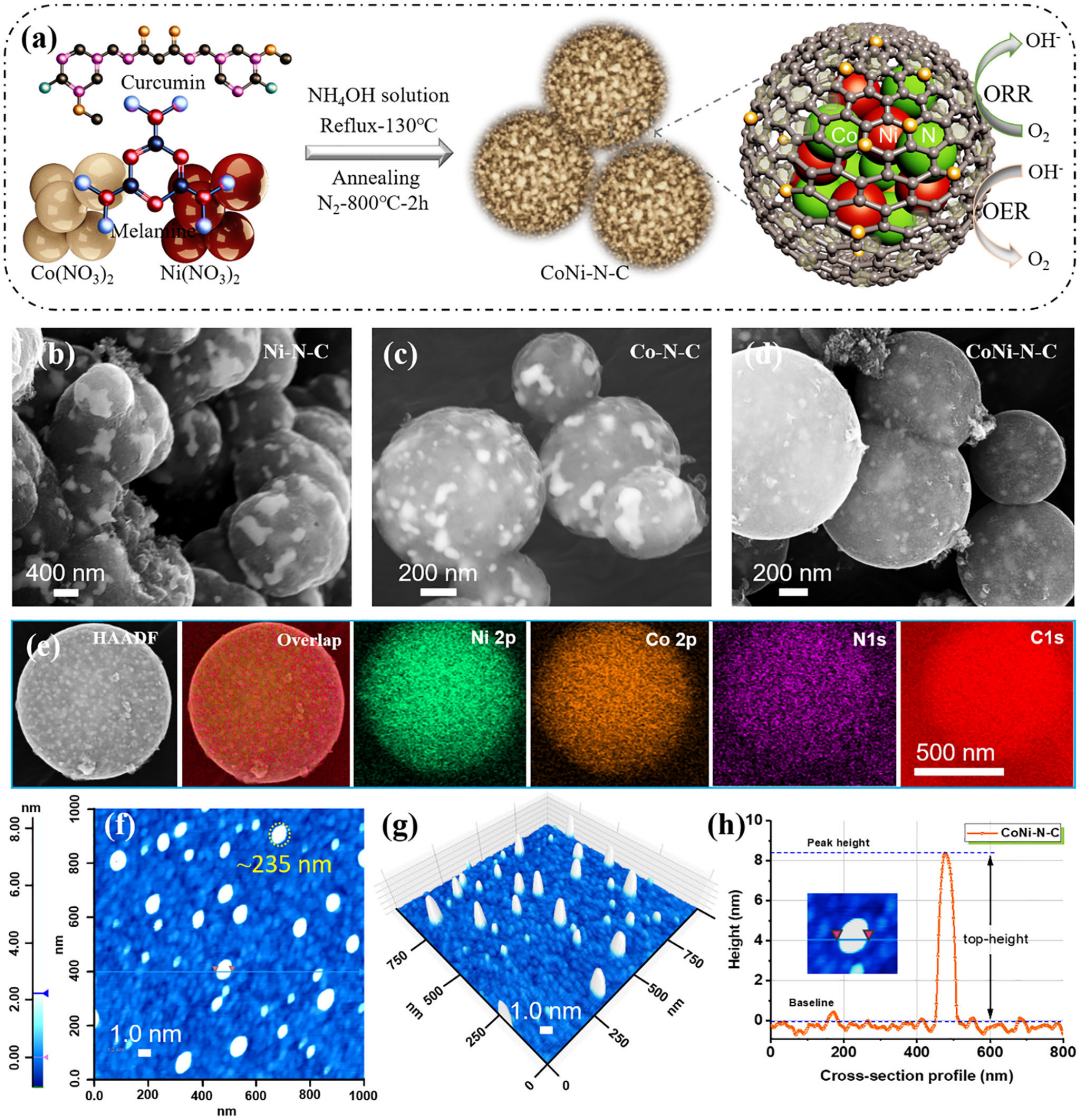
(a) Schematic illustration of the synthesis process of CoNi‐N‐C. SEM images of (b) Ni‐N‐C, (c) Co‐N‐C, (d) CoNi‐N‐C. (e) SEM‐EDS elemental mapping of CoNi‐N‐C. (f–h) AFM topographic results of CoNi‐N‐C.

The reflux technique was used to create CoNi alloy nanoparticles (NPs) anchored on extremely porous‐hybrid‐sphere structures (PHSs) in order to keep the reaction at a constant temperature without losing solvent. Curcumin and melamine have been preserved in an ethanol solution, as shown in Figure 1a. Then, while being agitated at room temperature, dropwise additions of cobalt (II) nitrate hexahydrate and nickel (II) nitrate hexahydrate and an ethanol solution were made. In the end, a tiny quantity of a solution of ammonium hydroxide was incorporated as a base catalytic reagent to produce curcumin‐melamine (C‐M), its called as San‐Yoo product, also known as hybrid‐metal–organic frameworks (Hybrid‐MOFs). The solution kept spinning and refluxing at 130°C even after being repeatedly cleaned with alcohol and DI water. As a result of association with transition metals such as cobalt (II) and nickel (II), dark brown powder is often seen in metal complexes instead of the free ligand. The powder, known as CoNi‐N‐C, was pyrolyzed for 2 h at a constant temperature of 800°C in an inert atmosphere. Ammonium hydroxide and ethanol are crucial components of this synthesis process because they assist in developing a hybrid‐sphere structure with CoNi in the internal regions as the outer layer carbon architect. As a solvent, ethanol helps create an intermediary material, such as a hybrid metal–organic framework nanotechnologies, which eventually becomes the precursor to porous carbon. Ammonium hydroxide tends to affect the organization of the intermediate compounds or the final porous carbons. Lastly, the synthesis of CoNi‐N‐C, Ni‐N‐C, and Co‐N‐C catalysts was examined using characterisation techniques.

The CoNi, Ni, and Co particles are homogeneous and have a porous‐hybrid‐sphere structure (PHSs) with a diameter of about 500 nm, according to scanning electron microscopy (SEM) images. The carbon pore diameters steadily rose, and the creation of porous carbon was maintained when the right number of atoms of carbon were present. In comparison to less porous materials (Ni‐N‐C and Co‐N‐C catalysts; Figure 1b,c), highly porous materials enhance mass mobility and electron transfer (CoNi‐N‐C catalysts; Figure 1d). Additionally, to create a mixture of Co and Ni NPs that occupy the porous region for particles with dimensions of around 235 nm, CoNi alloy nanoparticle structures were added to the highly porous carbon framework. Figures S1 and S2 depict the very porous‐hybrid‐sphere irregular Co and Ni nanoparticles as well as the porous framework of the interior layer of the Co‐N‐C and Ni‐N‐C catalysts. On another aspect, the exteriors of PHSs are smooth, and the particle size is identical when the CoNi hybrid structure occurs (Figure 1d).

The combined precise atomic percentages of CoNi‐N‐C, Ni‐N‐C, and Co‐N‐C PHSs as determined by high‐angle annular dark‐field (HAADF) imaging in conjunction with energy dispersive X‐ray spectroscopy (EDS or EDX) projections are displayed in Figure 1e; Figures S1 and S2a–f. Additionally, the elemental mapping of the Co, Ni, N, and C elements with the EDX spectrum in Figure S3a to further validate the fabrication of a CoNi‐N‐C catalyst. The presence of CoNi alloy NPs within the CoNi‐N‐C catalysts is indicated by a proportion of Co and Ni atoms that are equally apparent in the EDX spectrum; thus, it is directly related to the development of structures. The roughness of the surface of the CoNi‐N‐C catalyst was examined further utilizing atomic force microscopy (AFM); the results are shown in Figure 1f–h. 3D AFM scans of the catalyst show that the predicted surface cross‐section was around 235 nm, and the AFM images of the CoNi‐N‐C catalyst show that the CoNi NPs were anchored to highly porous hybrid spherical structures.

By increasing the absorption of target particle analytes on the CoNi‐N‐C catalyst's surface, this enhanced the electrochemical energy transfer applications. Figure S4a, b displays the N_2_ adsorption parameters employed in a Brunauer‐Emmett‐Teller (BET) study to quantify the surface area and porous characteristics of the Co‐N‐C and CoNi‐N‐C composites. In comparison to the Co‐N‐C composite (222.99 m^2^ g^−1^), the optimized CoNi‐N‐C had an enhanced specific surface of 306.32 m^2^ g^−1^. This research indicated the development of a highly porous hybrid‐carbon with metal particle catalyst at the optimal annealing temperature of 800°C. Barrett‐Joyner‐Halenda (BJH) modeling also showed variations in the CoNi NPs and Co NPs pore sizes in CoNi‐N‐C and Co‐N‐C‐based composites. The pore sizes of 2.80 and 2.51 nm in Figure S4b demonstrate that the Co and CoNi NPs were mesoporous. The CoNi‐N‐C composites wide pore dimensions and high particular surface area, along with their distinct mesoporous character, might enhance electron transfer and boost the quantity of active regions for energy conversion and storage. In contrast to Co‐N‐C and Ni‐N‐C, the overall structure of the CoNi‐N‐C PHSs composites exhibits stronger metal binding and a modified external framework.

**FIGURE 2:**
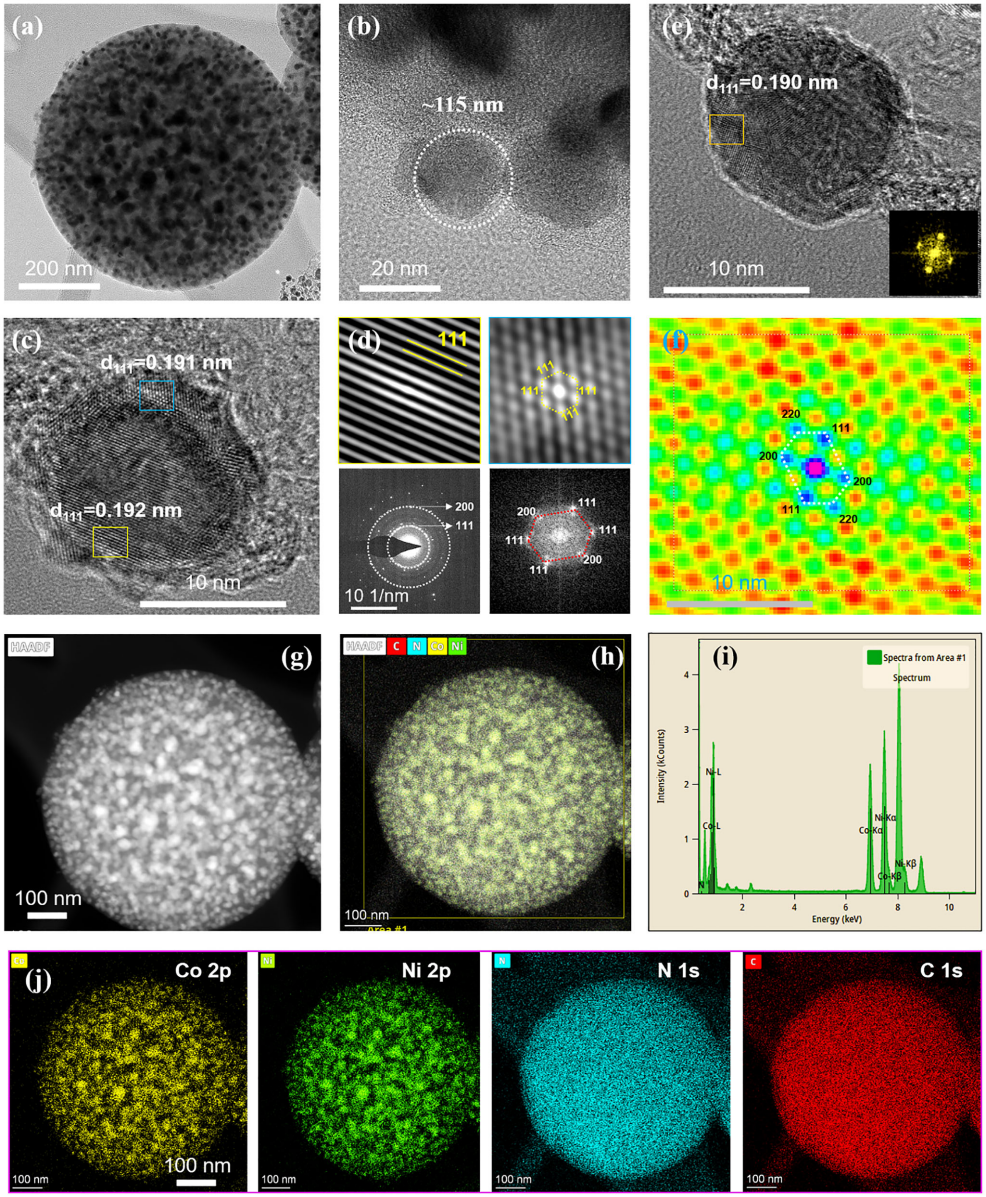
TEM analysis results of CoNi‐N‐C: (a,b) TEM, (c‐f) HR‐TEM, and (g) HAADF‐TEM image, (h) TEM‐EDS overlap image, (i) TEM‐EDS elemental spectra, and (j) TEM‐EDS mapping for the following Co 2p, Ni 2p, N 1s, and C 1s elements.

In addition to microscopy investigations of produced catalysts, Figure 2 displays the elemental mapping of CoNi‐N‐C as well as TEM (transmission electron microscopy), high‐resolution‐TEM, SAED (selected area electron diffraction), and HAADF‐STEM (high‐angle annular dark‐field scanning transmission electron microscopy) images. The material displayed a structure of carbon‐encapsulated CoNi alloy nanoparticles, with many CoNi‐N‐C PHSs extending outward from the porous carbon layers, as seen in Figures 2a and S5a,b. The inclusion of the curcumin‐melamine compound (San‐Yoo product) in Co and Ni metal fragments at high temperatures was thought to be the cause of the growth of these porous‐hybrid‐spheres. Reactant diffusion and charge transport efficiency were enhanced by this unique structure, which allowed internal active sites to be exposed to the electrolyte by PHSs stretching through the exclusive carbon layers. To accomplish to improve catalytic activity, CoNi‐N‐C PHSs also have a large specific surface area of 306.32 m^2^ g^−1^ (Figure S4a). In addition to boosting durability by shielding the active metal centers from electrolyte corrosion, these tiny porous hybrid sphere layers also improved electron transfer in comparison with conventional MOF‐based catalysts, which was advantageous for catalytic processes.

Interestingly, the spherical CoNi‐N‐C PHSs are encircled by a few CoNi alloy nanoparticles (NPs) with diameters of approximately 115 nm and lengths of several nanometers (Figures 2b and S5c). Additionally, lattice fringes analysis in Figures 2c,d and S5d showed an interplanar pixel spacing of 0.191 nm, which corresponds to the CoNi alloy NPs (111) diffraction plane, and a neighboring spacing of 0.182 nm, which is attributed to the CoNi (200) plane. Furthermore, the lattice fringes of Figure 2e,f show all important planes with hexagon order; these results supported the formation of a CoNi alloy. The presence of Co‐Ni alloy NPs in the metallic states of the CoNi‐N‐C PHSs is further confirmed by the SAED pattern in Figure 2d and the lattice characteristics in Figure S6a–c. The EDS elemental mapping and EDX spectrum in Figure 2g–j further established the presence of Co‐Ni atoms inside and C‐N atoms covering the exterior layer, demonstrating the efficient incorporation of hybrid MOFs‐derived CoNi‐N‐C PHSs catalysts. It further demonstrated, consistent with the XRD data, the existence of the specified crystalline phases and elements.

**FIGURE 3:**
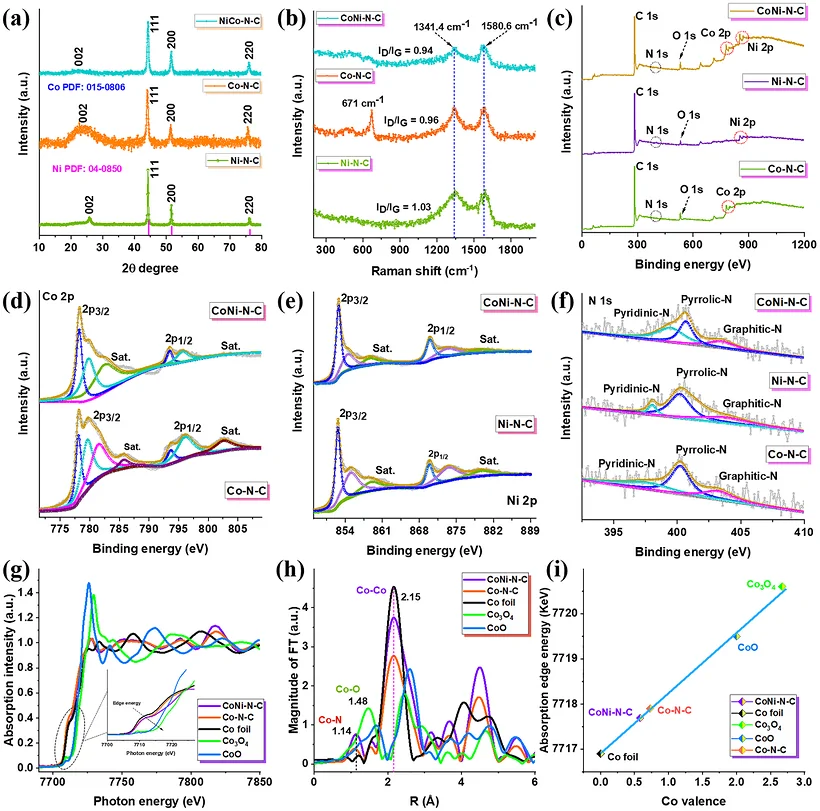
(a) XRD and (b) Raman analysis results of Ni‐N‐C, Co‐N‐C, and CoNi‐N‐C composites. XPS spectrum of (c) survey spectrum, (d) Co 2p, (e) Ni 2p, (f) N 1s. XAS analysis: (g and h) XANES and EXAFS *R*‐space spectra of Co *k*‐edges for CoNi–N–C, Co‐N‐C, commercial Co_3_O_4_, CoO, and Co foil. (e) The average oxidation state of Co in Co_3_O_4_, CoNi‐N‐C, and Co‐N‐C is determined by the correlation between K‐edge energy and Co oxidation state. A solid line indicates a linear relationship based on reference samples.

The crystal structures of the catalysts were investigated using X‐ray diffraction (XRD) analysis. Ni‐N‐C, Co‐N‐C, and CoNi‐N‐C had distinct peaks caused by at 25.7°, 23.8°, and 22.2°, which are possibly indexed to the 002 planes of graphitic carbon since hybrid‐MOFs are fully transformed into activated carbon at 800°C, as seen in Figure 3a. Furthermore, the (111), (200), and (220) planes of crystals of the aforementioned Ni‐N‐C, Co‐N‐C, and CoNi‐N‐C catalysts with appropriate Ni and Co JCPDS cards for Ni‐PDF 04–0850; Co‐PDF 015–0806 were assigned significant refractions at 44.2°, 51.5°, and 76.1° [33]. These findings supported the successful production of CoNi nanoparticles by confirming a combination of CoNi mixture and Co‐N as well as Ni‐N phases [34]. Furthermore, it should be mentioned that all of the peaks in the materials synthesized that correspond to the diffraction of Co or Ni are shifted to the center of the standard location of metallic cobalt and metallic nickel reported in the database, indicating the formation of Co‐Ni alloy nanoparticles [33, 35]. As a result, it enhanced the catalytic performance by encouraging increased exposure of active sites.

The Raman spectra in Figure 3b showed distinct D and G bands at about 1341.4 and 1580.6 cm^−1^, respectively, which corresponded to the lattice defects and degree of graphitization of the material in carbon atoms. The defect mass in the structure of carbon was immediately reflected by the magnitude ratio of D and G bands (I_D_/I_G_) [36]. The corresponding I_D_/I_G_ ratios for Ni‐N‐C, Co‐N‐C, and CoNi‐N‐C were 1.03, 0.96, and 0.94, respectively, suggesting that CoNi‐N‐C had a lower defect content and a higher degree of graphitization. This is likely due to the etching impact of nickel following the carbonization process as well as the greater microporosity shown by sorption isotherms [33].

The catalysts valence states and chemical structure were discovered by X‐ray photoelectron spectroscopy (XPS). The existence of Co, Ni, N, and C elements in Ni‐N‐C, Co‐N‐C, and CoNi‐N‐C composites was verified by the XPS survey spectrum (Figure 3c). The Co 2p spectrum of CoNi‐N‐C in Figure 3d had satellite peaks at 785.8 and 802.7 eV, which were linked to Co agitation characteristics; peaks at 779.2 and 794.5 eV, which were attributed to the metallic Co^0^ in the CoNi‐N‐C and Co‐N‐C; and peaks at 778.3 and 794.6 eV, which were assigned to the oxidized Co species in CoNi‐N‐C [37]. The Co‐N‐C composite metallic peak intensity is lower relative to that of CoNi‐N‐C due to persistent particle growth in CoNi alloy NPs. The Ni 2p spectrum of CoNi‐N‐C showed peaks at 852.7 and 870.0 eV, which corresponded to Ni^3+^ in the CoNi alloy and Ni nanoparticles; peaks at 854.5 and 873.2 eV, which were linked to cationic Ni species; and peaks at 858.6 and 879.2 eV, which were attributed to Ni satellite signals, as illustrated in Figure 3e [38]. Interestingly, the existence of CoNi alloy nanoparticles is confirmed by the integration of Ni^0^ and Co^0^, which is consistent with the XRD data [39].

The pyridinic‐N, pyrrolic‐N, and graphitic‐N in CoNi‐N‐C were represented by peaks in the N 1s spectra (Figure 3f) at 399.4, 400.6, and 403.4 eV, respectively. Pyridinic‐N and pyrrolic‐N are the predominant N species in CoNi‐N‐C and are thought to be significant factors in high ORR activity; concurrently, the Co‐N‐C and Ni‐N‐C composite exhibits high pyrrolic‐N species [40]. The C 1 s spectra revealed the existence of C─C (284.0 eV), C─N (285.0 eV), and C─O (288.8 eV) bonds in CoNi‐N‐C, as seen in Figure S7a. The chemical composition and binding energies of C were virtually unchanged before and after Co‐Ni doping. The advantageous creation of the CoNi‐N‐C hollow sphere structure was responsible for the binding affinities in CoNi‐N‐C shifting toward lower values overall when compared to Co‐N‐C and Ni‐N‐C (Figure S7b). The materials electrical configuration was optimized by this hollow sphere structure, and the alloy and metal nitrides synergistic interaction improved interfacial charge transfer, raised the total electron cloud density, and ultimately decreased the binding energy (Figure S7d) [41].

X‐ray absorption near‐edge structures (XANESs) and extended X‐ray absorption fine structures (EXAFSs), which are extremely sensitive instruments for identifying the active species in catalysts, were recorded in order to investigate the local electronic and geometric structures of the prepared CoNi‐N‐C and Co–N–C catalysts. As seen in Figure 3g, the Co K‐edge XANES spectra of CoNi‐N‐C and Co–N–C were collected in order to investigate the charge state of Co. A mixed‐valence state of Co^2+^/Co^3+^ was indicated by the pre‐edge peak characteristic of the Co K‐edge of Co‐N‐C and CoNi‐N‐C, which was similar to that of the Co_3_O_4_ standard reference. The pre‐edge and absorption edge positions of CoNi–N–C, however, showed different characteristics from those of commercial Co_3_O_4_. They shifted to a lower energy position, indicating a decrease in the oxidation state of Co and the successful production of Co–N–C and CoNi–N–C composites. Additionally, the Co R‐space FT‐EXAFS spectra of Co‐N‐C (1.10 and 2.15 Å) and CoNi‐N‐C showed strong peaks at roughly 1.14 and 2.15 Å, respectively, which were linked to Co–N and Co–Co coordination (Figure 3h), demonstrating significant N–C interactions with Co ions and charge transmission to the metal.

The aforementioned observation is further supported by Figure S7c, which displays different EXAFS oscillations for the CoNi‐N‐C and Co–N–C catalysts. This suggests that the coordination environments surrounding the Co ions are different, and the decrease in oscillation amplitude in Co–N–C suggests the presence of a more disorganized structure. The substrate‐normalized absorption edge examines the Co atoms' valence state (Figure 3i). By examining the absorption edge location or the first variations of various XANES spectra, one can typically ascertain the average oxidation state. Yoo et al. and Daqin Guan et al. also used a similar technique to determine the valence state of the materials that were synthesized [42, 43]. According to the expected valence state, the amount of Co in CoNi–N–C turned out to be much lower than that in Co‐N‐C, suggesting a more constrained electronic environment. The combined Co^2+^/Co^3+^ states in Co‐N‐C yielded an average valence of about +2.7 with a larger absorption edge. On the other hand, CoNi–N–C showed an absorption edge location close to Co foil (Co^0^) because of strong Co–N coordination. This implies that electrons from the N‐doped graphite lattice are donated to stabilize electron‐rich Co centers. Figure S7d, based on XAS and XRD analysis, confirmed the electronic structure.

### 3.2 Electrochemical Performances of Ni‐N‐C, Co‐N‐C, and CoNi‐N‐C Electrocatalysts

The ORR and OER are fundamental processes in electrochemistry and play an important role in renewable energy technologies such as metal–air batteries and fuel cells. Nevertheless, their practical application is limited by sluggish kinetics and significant overpotentials. ORR is responsible for oxygen reduction during discharge, while OER governs oxygen evolution during the charging process. Consequently, the development of efficient ORR and OER catalysts is critical for advancing electrochemical energy conversion and storage devices. To evaluate the bifunctional oxygen electrocatalytic performance was first examined through ORR studies using Ni–N–C, Co–N–C, and CoNi–N–C electrocatalysts. Cyclic voltammetry (CV) measurements were carried out with 0.1 M KOH electrolyte under N_2_ and O_2_‐saturated conditions to investigate their ORR activity. As presented in Figure S8, no noticeable reduction peak was observed in the N_2_‐saturated electrolyte, while clear cathodic reduction peaks appeared after O_2_ saturation, confirming oxygen reduction activity. The position and intensity of these cathodic peaks indicate the ORR electrocatalytic performance of the catalysts. Among the prepared samples, CoNi–N–C demonstrated enhanced ORR activity with a more positive peak potential of approximately 0.781 V, outperforming most reported bio‐carbon‐based catalysts.

**FIGURE 4:**
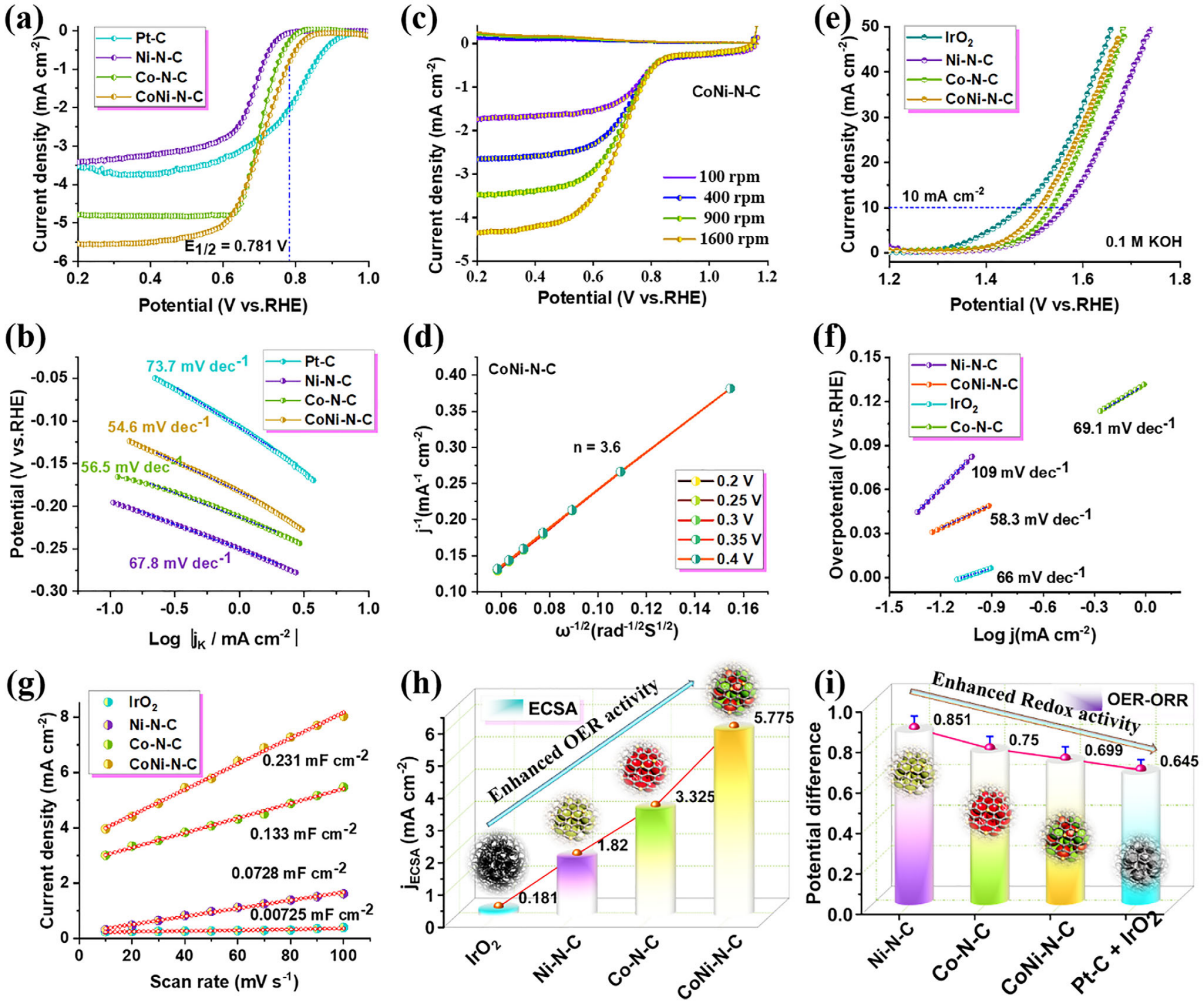
ORR performance of prepared catalysts in 0.1 M KOH: (a) LSV curves at scan rate 10 mV, (b) Tafel slopes results of Pt‐C, Ni‐N‐C, Co‐N‐C, and CoNi‐N‐C electrocatalysts. (c) LSV curves in a rotating ring‐disk electrode (RRDE) setup, (d) K‐L plots of CoNi‐N‐C electrocatalyst. OER performance of prepared catalysts in 0.1 M KOH: (e) LSV at scan 1 mV, (f) Tafel slopes, (g) C_dl_ values, (h) ESCA values, and (i) potential differences (ΔE = E_OER_‐E_ORR_) of Pt‐C, Ni‐N‐C, Co‐N‐C, and CoNi‐N‐C electrocatalysts.

Further, in order to evaluate the ORR activity, the linear sweep voltammetry (LSV) measurements were performed using a rotating disk electrode (RDE) at 1600 rpm in O_2_‐saturated 0.1 M KOH solution of the prepared catalysts (Figures 4a and S9a–d). The background current obtained under N_2_ atmosphere was subtracted to correct the current density. Among the tested samples, CoNi–N–C exhibited slightly improved ORR performance with an onset potential (E_onset_) of 0.917 V and a half‐wave potential (E_1/2_) of 0.781 V, which can be attributed to the formation of well‐dispersed nanoparticles that provide abundant and stable active sites for oxygen reduction (Table S1). In comparison, Co–N–C (E_onset_ = 0.849 V, E_1/2_ = 0.733 V) and Ni–N–C (E_onset_ = 0.845 V, E_1/2_ = 0.715 V) showed lower activity, while the performance of CoNi–N–C approached that of commercial Pt/C (E_onset_ = 0.95 V, E_1/2_ = 0.87 V). Furthermore, CoNi–N–C delivered a higher limiting current density (5.0 mA cm^−2^) than Pt/C (4.16 mA cm^−2^), indicating enhanced ORR kinetics.

Tafel slope analysis (Figure 4b) was further conducted to investigate the reaction kinetics and possible electrochemical mechanism. CoNi–N–C showed a relatively small Tafel slope (54.6 mV dec^−1^), suggesting faster reaction kinetics than Co–N–C (56.5 mV dec^−1^), Ni–N–C (67.8 mV dec^−1^), and Pt/C (73.7 mV dec^−1^). LSVs recorded at different rotation speeds (400–2800 rpm) were used to investigate the electron transfer behavior of the catalysts during ORR (Figure S8a–d). The corresponding Koutecky–Levich (K–L) plots for Ni–N–C, Co–N–C, and CoNi–N–C are presented in Figures 4d and S10a–d, respectively, showing a gradual increase in current density with increasing rotation speed within the potential range of 0.3–0.6 V. All catalysts exhibited good linearity and nearly parallel K–L plots, comparable to commercial Pt/C, indicating first‐order reaction kinetics with respect to dissolved oxygen. Furthermore, LSV curves at rotation speeds of 400–2800 rpm were recorded to 4‐electron reaction pathways using a rotation ring disk electrode (Figure 4c,d).

According to the K–L plot and LSV curves using RRDE, CoNi–N–C demonstrated an electron transfer number of approximately 3.6 e^−^, which is higher than Ni–N–C (2.2 e^−^), Co–N–C (3.4 e^−^), and Pt‐C (3.5 e^−^), suggesting a dominant four‐electron ORR pathway. The formation of H_2_O_2_ as an intermediate and its possible conversion to H_2_O can occur through disproportionation (2H_2_O_2_ → O_2_ + 2H_2_O) or electrochemical reduction (H_2_O_2_ + 2H^+^ + 2e^−^ → 2H_2_O), which may contribute to the apparent four‐electron pathway. Repeated reduction cycles of oxygen molecules can therefore lead to an overall four‐electron reduction process, even when small amounts of H_2_O_2_ are formed. Crucially, CoNi–N–C demonstrated exceptional methanol tolerance: when 1.0 M methanol was added, the relative ORR current was constant while Pt/C lost more than half of its electrical activity (Figure S11) [43]. To investigate the durability of CoNi–N–C, ORR cyclic stability was performed for 10,000 cycles at a scan rate of 500 mV s^−1^ (Figure S10b). After long‐term cycling, a noticeable decrease in current density was observed in LSV curves, whereas E_onset_ potential remained nearly unchanged, confirming the excellent electrochemical stability and structural robustness of the catalyst.

The OER performance of the prepared catalysts was evaluated using a three‐electrode system consisting of a graphite rod as the counter electrode, Ag/AgCl as the reference electrode, and Ni–N–C, Co–N–C, and CoNi–N–C as working electrodes coated on carbon paper. CV measurements were conducted in 0.1 M KOH electrolyte at a scan rate of 50 mV s^−1^ to examine their electrochemical behavior (Figure S11). The CV curves of Ni–N–C, Co–N–C, CoNi–N–C, and IrO_2_ displayed clear oxidation and reduction features, indicating active electrochemical processes. LSV results (Figure 4e) revealed that CoNi–N–C exhibited improved OER performance with a lower overpotential at 10 mA cm^−2^ (Eη_10_ = 1.51 V) compared to Ni–N–C (1.558 V) and Co–N–C (1.535 V), while showing performance comparable to the benchmark IrO_2_ catalyst (1.49 V). A comparison with previously reported CoNi‐based catalysts (Table S1 and Figure S15a) further confirms that the incorporation of Co and Ni into the CoNi–N–C structure enhances electrocatalytic activity, leading to improved OER performance relative to other bio‐carbon‐supported CoNi systems. The OER reaction kinetics were further evaluated using Tafel slope analysis (Figure 4f). The Tafel slopes of Ni–N–C, Co–N–C, CoNi–N–C, and IrO_2_ were calculated to be 109, 69.1, 58.3, and 66 mV dec^−1^, respectively. Based on these Tafel slope values, Co–N–C exhibited a relatively low Tafel slope, indicating faster OER kinetics among the prepared catalysts and IrO_2_, while Ni–N–C and CoNi–N–C showed comparatively higher slopes, suggesting slower reaction kinetics. Electrochemical impedance spectroscopy (EIS) was further conducted to investigate the charge‐transfer behavior of the catalysts (Figure S12a,b). The charge‐transfer resistance (Rct) values were determined to be 5.75 Ω for Ni–N–C, 3.02 Ω for Co–N–C, 1.88 Ω for CoNi–N–C, and 6.7 Ω for IrO_2_, indicating improved electrical conductivity and faster interfacial electron transfer for CoNi–N–C compared to other prepared catalysts and IrO_2_. The EIS data were fitted using an equivalent circuit model (Figure S13), which represents the electrochemical processes occurring at the electrode–electrolyte interface. The model includes series resistance (Rs), charge‐transfer resistances (R_1_ and R_2_), and constant‐phase elements (Q_1_ and Q_2_) corresponding to the counter and working electrodes. The Rs value mainly originates from the contact resistance between the electrocatalyst and the current collector (carbon paper), while the lower Rct values of CoNi–N–C suggest efficient charge transport, enhanced conductivity, and improved OER reaction kinetics.

The origin of the enhanced OER performance was further investigated by estimating the electrochemically active surface area (ECSA) from CV measurements conducted at different scan rates in the non‐Faradaic region to determine the double‐layer capacitance (C_dl_) of the prepared catalysts (Figure S14a–d). As presented in Figure 4g, the C_dl_ values of Ni–N–C, Co–N–C, CoNi–N–C, and IrO_2_ were calculated to be 0.0728, 0.133, 0.231, and 0.00725 mF cm^−2^, respectively. The corresponding ECSA values (Figure 4h) indicate that CoNi–N–C possessed the largest electrochemically active surface area, followed by Co–N–C, Ni–N–C, and IrO_2_, suggesting a higher number of accessible active sites in CoNi–N–C. However, OER performance is influenced not only by ECSA but also by intrinsic catalytic activity, electrical conductivity, and metal–support interactions.

The durability of the CoNi–N–C electrocatalyst was further examined using multi‐step chronopotentiometry at different current densities (Figure S15). The CoNi–N–C catalyst showed outstanding stability, as evidenced by a minimal potential shift of only 31 mV between initial and post‐stability LSV curves, demonstrating durable OER performance. The bifunctional ORR/OER performance of the CoNi–N–C electrocatalyst was evaluated using the potential difference (ΔE), which is commonly used to assess dual‐functional oxygen electrocatalytic activity. Generally, a smaller ΔE value indicates better bifunctional performance. Based on the ORR and OER polarization curves (Figure 4i; Figures S16a,b and Table S1), CoNi–N–C exhibited a ΔE value of 0.699 V, which was significantly lower than those of Ni–N–C (0.851 V) and Co–N–C (0.75 V), demonstrating improved bifunctional activity. Although the benchmark Pt/C + IrO_2_ catalyst showed a lower ΔE value of 0.645 V, CoNi–N–C displayed competitive performance among the prepared materials.

### 3.3 Synthesis and Evaluation of CoNi‐BTC MOF as a Coating Composite

**FIGURE 5:**
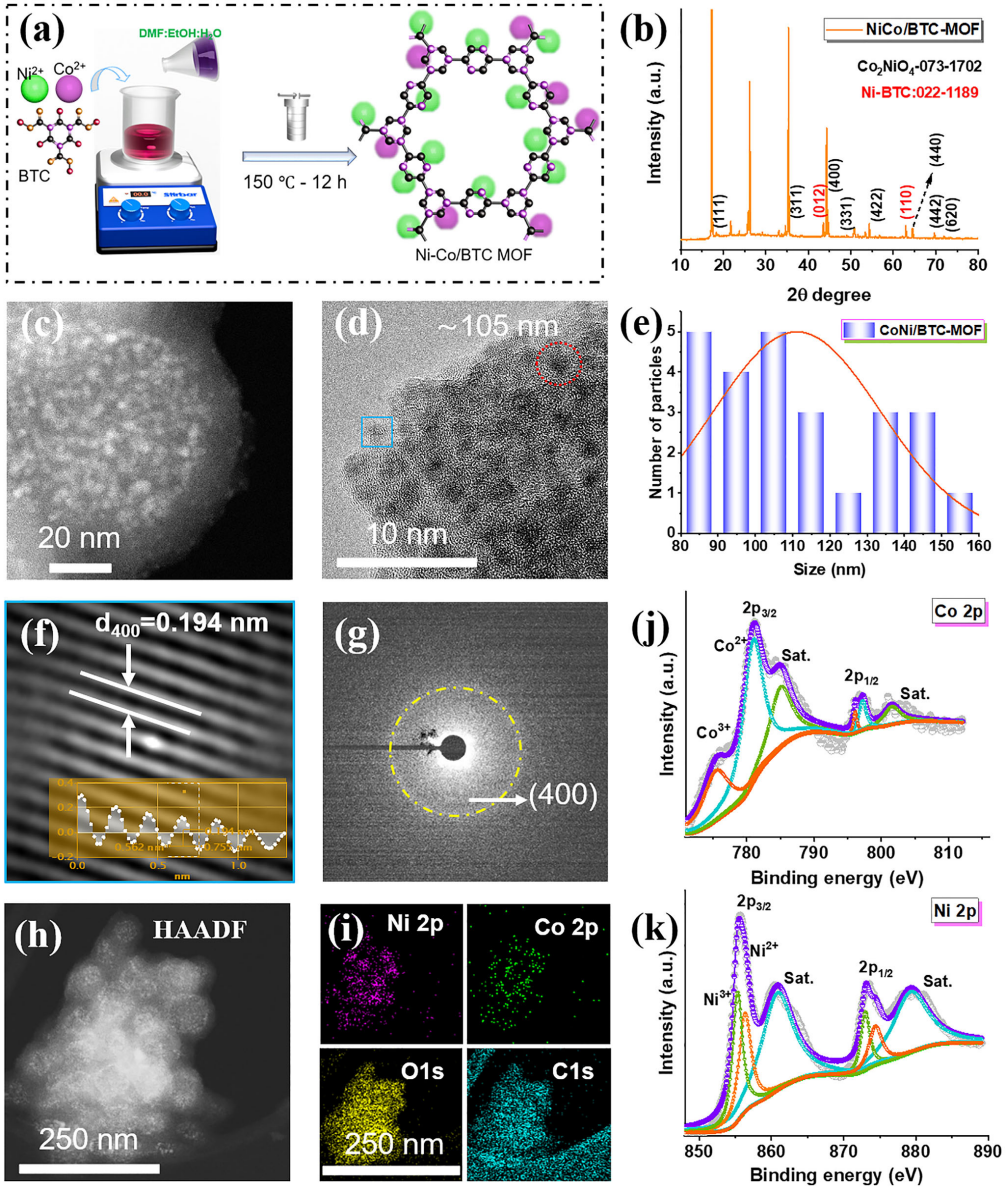
Characterization results of NiCo‐BTC MOF: (a) Synthesis route, (b) XRD, (e,d) TEM, (f) HR‐TEM, (g) SEAD, (h,i) TEM‐EDS elemental mapping, (e) bar‐chart of average particle size distribution. High‐resolution XPS spectra of (j) Co 2p and (k) Ni 2p.

Figure 5a illustrates the hydrothermal synthesis of CoNi NPs with combined BTC‐MOF for the creation of CoNi‐BTC MOFs composites, which was verified by FT‐IR, XRD, SEM, TEM, and XPS analyses. The abundance of hydroxyl molecules and C‐H stretching vibrations is responsible for the broad bands at 3470.5 cm^−1^ and 3093.5 cm^−1^ in the FT‐IR (Fourier transform‐infrared spectroscopy) of the CoNi‐BTC MOF composite (Figure S17a,b), that belong unique to Ni‐Co MOF. The peaks at 722.5 and 1615.5 cm^−1^ represent the C‐H and C‐C vibrations. The notable transmittance seen at 1439.2 cm^−1^ is connected with the C‐O stretching vibration. Ni‐Co MOF displays two peaks at 1370 and 1552 cm^−1^ upon contact, which are ascribed to symmetric and asymmetric bending modes as well as Ni─OH, Co─OH, or NiCo─OH bonds.

The XRD data of CoNi‐BTC MOFs are shown; the peaks at 2θ = 18.45° and 36.7° correspond to the (111 and 311) depiction of the CoNi‐BTC composite (Figure 5b). CoNi‐BTC composite characteristic peaks match the literature exactly. The patterns from XRD of CoNi‐BTC MOFs hybrids show the distinctive BTC peak, which could be caused by the following: (i) The combination state and neatness of layers of BTC are reduced by the increased consistency of CoNi distribution on the BTC surface. (ii) Its delicate distinctive peak is caused by relatively low BTC dosage. CoNi‐BTC composites are ground to totally break the Ni‐BTC crystal in order to detect the presence of BTC. The resulting composite materials are then subjected to another XRD characterization with suitable JCPDS cards: 022–1189 (Ni‐BTC) and 073–1702 (Co_2_NiO_4_); it is evident that the distinctive peak of CoNi‐BTC occurs. This is compelling evidence that BTC exists in CoNi‐BTC. Because too much BTC reduces the crystallinity of Ni‐BTC, the peak features of CoNi‐BTC are less visible in the BTC XRD pattern.

SEM images are displayed in Figure S18 to provide further visual characterization of the shape of the as‐prepared composites. The diameters of CoNi nanoparticles on BTC‐MOF are approximately 0.1 µm. These include homogeneous spheres with smooth surfaces (Figure S18a). The sphere‐like shape of the CoNi nanoparticles in comparison to CoNi‐BTC indicates that BTC has little impact on the framework of CoNi‐BTC. Similar phase transitions and noticeably smoother surfaces are seen in the composites. Furthermore, the morphology of the composites is compatible with the SEM image of BTC, and it is evident that the surface is coated with layered frameworks (Figure S17b). The homogeneous distribution of Co, Ni, O, and C elements on the composite surface is further demonstrated by the EDS images and EDS spectra of CoNi‐BTC (Figure S18c–e). Transmission electron microscopy view in Figure 5c–i, which is comparable to the SEM results, reveals that the CoNi nanoparticle structure is present inside the carbon layer with a sphere shape and a particle size of around 105 nm (Figures 5c–e and S19a,b). The d‐spacing value of the typical crystalline plane of (400) in CoNi_2_O_4_ corresponds to the observed d‐spacing value of CoNi, which is 0.194 nm (Figure 5f,g). While the elemental map results (Figure 5h,i) reveal the uniform distribution of the aforementioned elements, the EDS spectrum data (Figure S19c) demonstrate the coexistence of Co, Ni, O, and C in CoNi‐BTC.

XPS spectra were obtained to determine the catalyst valence states and chemical structure of the catalysts. The XPS scan spectra for CoNi‐BTC MOFs show the presence of Ni 2p, Co 2p, C1s, and O 1s signals, as shown in Figure S20a. When paired with the Co^2+^ state, which displays XPS peaks at 781.1 (2p_3/2_) along with 797.1 eV (2p_1/2_) in the CoNi‐BTC MOFs composite, the XPS spectra of Co 2p exhibit peaks at 780.2 and 796.1 eV in Figure 5j, corresponding to Co 2p_3/2_ and Co 2p_1/2_ in the CoNi‐BTC composite, respectively. According to the literature, the CoNi‐BTC MOFs composite Ni 2p spectra revealed Ni 2p_3/2_ and Ni 2p_1/2_ at 855.5 and 873.3 eV, respectively, indicating the presence of Ni^2+^/Ni^3+^ in this composite (Figure 5k). Furthermore, in contrast to reference spinel catalysts, the XPS peak binding energy for Co^2+^/Co^3+^ states was marginally modified following the combination of Co‐Ni elements in the BTC‐MOFs composite [44, 45]. CoNi‐BTC MOFs composites O 1s and C 1s spectra were also thoroughly investigated in the 531.5 and 285.2 eV areas in Figure S20b,c.

### 3.4 Performance of Zn‐Air Cell

**FIGURE 6:**
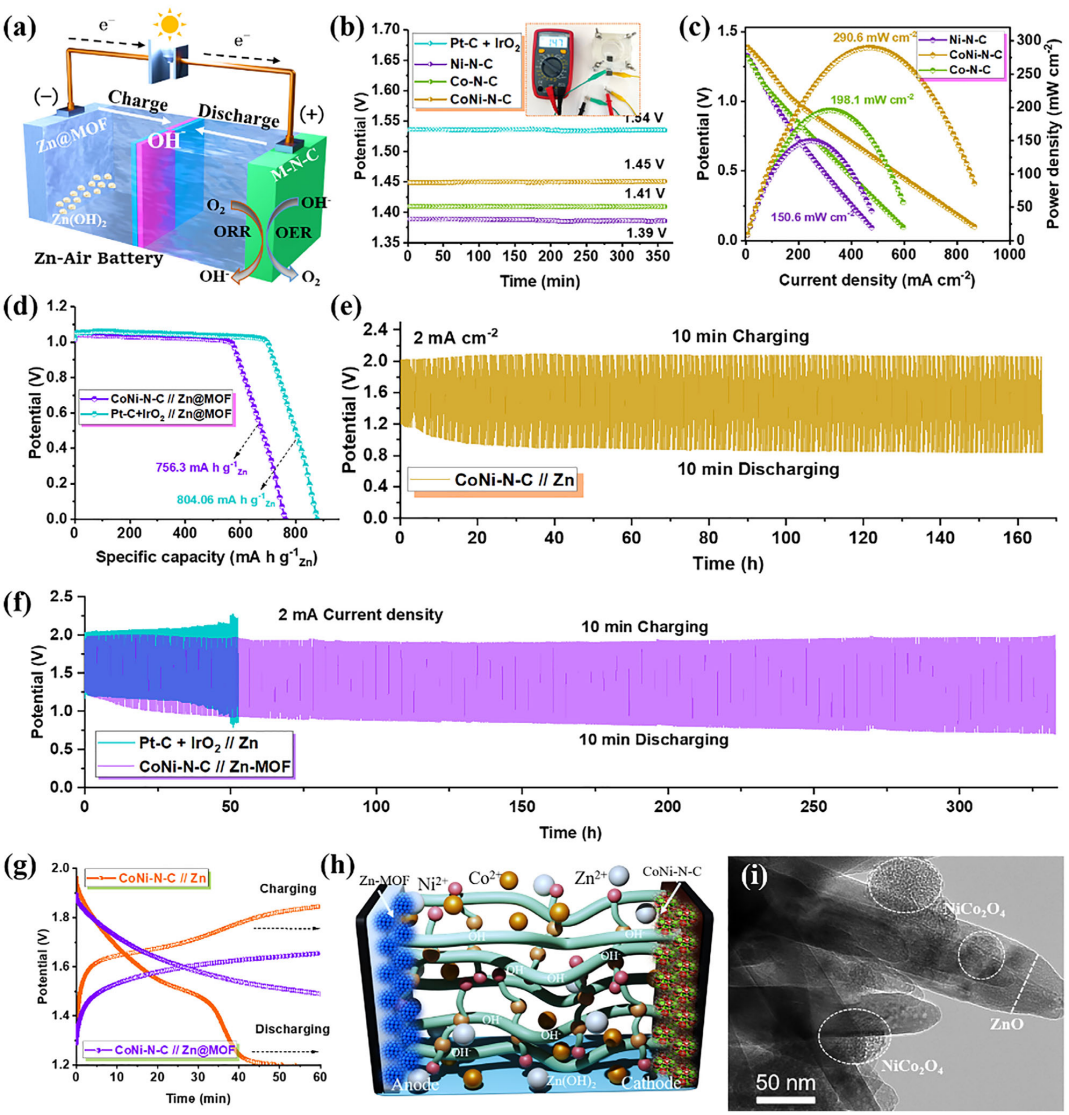
Zinc‐air battery performance of prepared catalysts in 6 M KOH with 0.2 M zinc acetate electrolyte: (a) Illustrative image of ZAB setup, (b) OCV, (c) polarization curve, (d) specific capacity of Pt‐C + IrO_2_ // Zn@MOF and CoNi‐N‐C//Zn@MOF electrodes. (e) Charge–discharge cycle of CoNi‐N‐C//Zn: 10 min charging, 10 min discharging at 2 mA current density. (f) Charge–discharge cycle of Pt‐C+IrO_2_//Zn and CoNi‐N‐C//Zn@MOF, (g) charge–discharge curve of CoNi‐N‐C//Zn and CoNi‐N‐C//Zn@MOF, (h) schematic illustration of a ZAB based on a Zn‐MOF anode and CoNi‐N‐C air cathode, (i) TEM image of Zn‐MOF//Zn electrode.

Excellent ORR/OER bifunctional catalytic characteristics were demonstrated by the Ni‐N‐C, Co‐N‐C, and CoNi‐N‐C. Its utility was validated by building a ZABs. An electrolyte metal‐plate anode and an air‐electrode cathode are the main parts of a ZABs. Figure 6a shows an overall schematic of the ZABs. The zinc plate on the anode undergoes electrochemical oxidation, producing electrons that cause a current to flow. Catalytic ORR takes place on the cathode at the airflow electrode in the ZABs core [29, 46]. First, the breathable layer of the air electrode absorbs oxygen from the atmosphere's air. After diffusing to the catalytic layer, it joins the electrolyte and initiates a three‐phase ORR reaction (Overall reaction: Zn + ½ O_2_ ⇌ ZnO).

**FIGURE 7:**
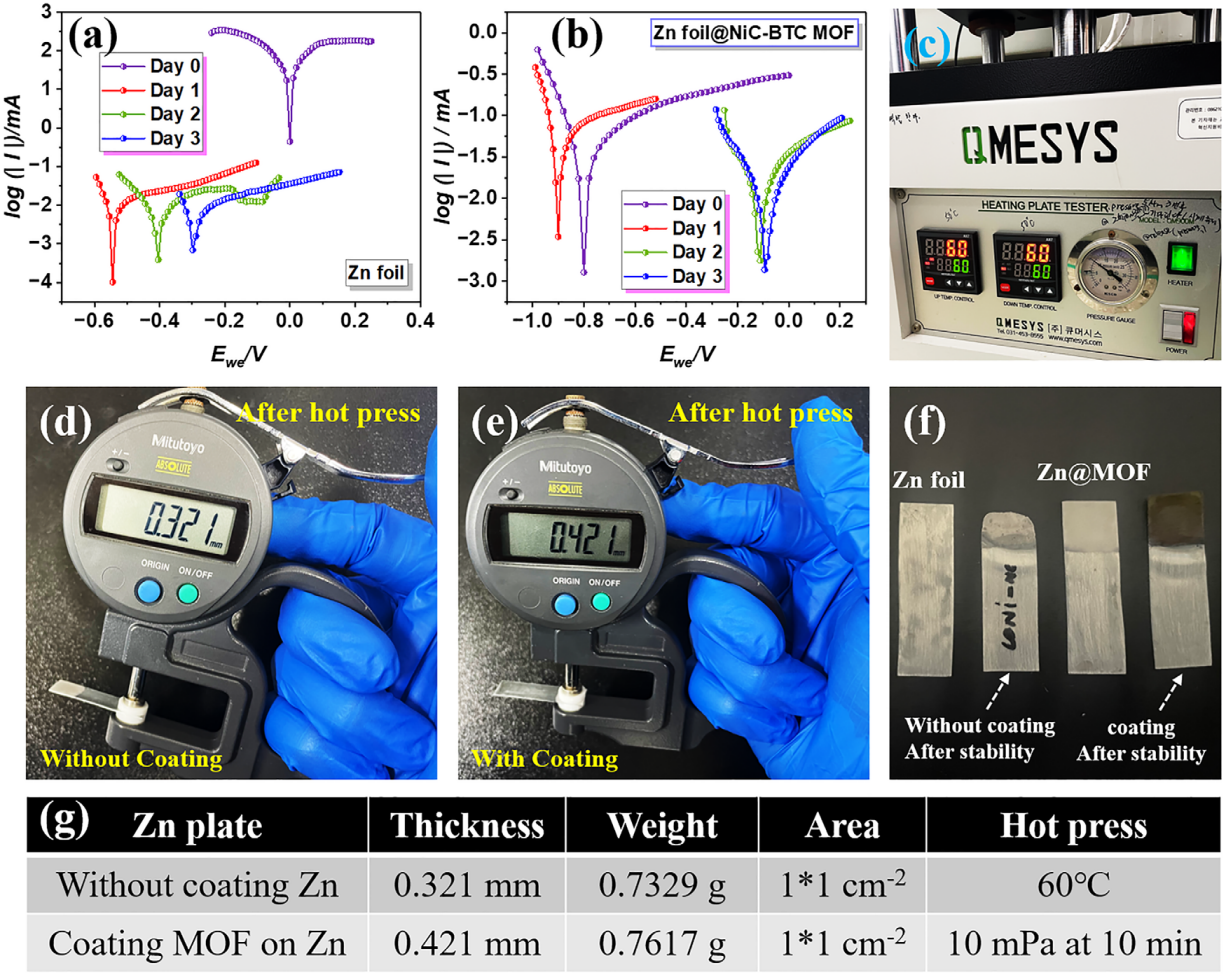
Tafel plots of (a) bare zinc (b) CoNi‐MOF@Zn electrodes in aqueous electrolyte as a function of time. (c) Heating plate tester, digital thickness gauge (d) without coating Zn plate, (e) NiCo‐MOF coating on Zn plate, (f) Zn‐air battery Zn anode electrode, and (g) table of measurement conditions for zinc plates.

When measuring voltage in circuits for electricity or electronic devices, a voltmeter was employed. The rise in oxygen content in ZABs with a CoNi‐N‐C electrocatalyst and a voltage of ∼1.47 V for two ZABs linked to a voltmeter is displayed in Figure 6b (insert image). A light‐emitting diode (LED) with a rated voltage of ≈2.90 V was powered by dual liquid‐state ZABs linked in series (Figure S21a,b). For 360 min, an open‐circuit voltage (OCV) of ∼1.45 V was measured and held steady (Figure 6b). The maximum power density of Ni‐N‐C (150.6 mW cm^−2^), Co‐N‐C (198.1 mW cm^−2^), and CoNi‐N‐C (290.6 mW cm^−2^) was considerably closer than that of Pt/C + IrO_2_, according to the enhanced OER/ORR behavior (Figure 6c). The specific capacity is subsequently determined using galvanostatic studies at 10 mA cm^−2^ based on the mass decrease of zinc plates. The ZAB based on CoNi‐N‐C has a specific capacity of 724.9 mA h g^−1 ^_Zn_, which is comparable to the batteries based on Pt/C + IrO_2_ // Zn (782.1 mA h g^−1 ^_Zn_) and Pt/C + IrO_2_ // Zn@MOF (804.06 mA h g^−1 ^_Zn_) in Figures 6d and S21c. Following the coating of CoNi‐BTC MOFs on the Zn anode, the specific capacity gradually increased to 756.3 mA h g^−1 ^_Zn_ at 10 mA cm^−2^. The result was higher than that of bare Zn foil because the CoNi‐BTC@Zn electrode was more efficient and leached smaller amounts of Zn. Figure 7f illustrates the high precipitation of Zn foil and the low precipitation following the coating of CoNi‐BTC MOFs on Zn foil. Because of its affordability and environmental friendliness, this highly hybrid‐MOFs‐assisted cathode and MOFs‐derived anode Co‐Ni NPs catalyst performed better in a ZAB than a recently reported catalyst (Table S1).

Figure 6e displays the initial charge–discharge voltage difference and round‐trip efficiency of the CoNi‐N‐C // Zn‐based ZABs. Additionally, battery cyclability was evaluated utilizing a high rate of current over a 166 h cycle that comprised 10 min of charging and 10 min of discharging at a current density of 2 mA cm^−2^. Following CoNi‐BTC MOFs coated on Zn foil (Figure 6f), CoNi‐N‐C // CoNi‐BTC@Zn‐based ZABs show 333.33 h of cycle stability with reduced potential differences because CoNi‐BTC MOFs inhibit Zn foil degradation with high concentrated electrolyte (6 M KOH + 0.2 M Zn(OAc)_2_). With enhanced return efficiency from the starting (44%) to the final (39.5%) after 500 cycles, the CoNi‐N‐C // Zn Zn‐air battery exhibits good charge–discharge stability. However, after 1000 cycles, CoNi‐N‐C // CoNi‐BTC@Zn‐based ZABs exhibit lower potential differences (0.99 to 1.22 V) and lower round‐trip efficiency (50.0%) compared to the final (39.2%) (Figure 6g). These results convincingly show that a reusable ZAB with enhanced performance could be assembled using CoNi‐N‐C as the air cathode and Zn vs modified Zn foil as the anode (Table S1). Additionally, the charge–discharge stability of the CoNi‐BTC‐coated Zn anode using Pt‐C+IrO_2_ cathode electrodes (5 mA current density). Because CoNi‐BTC MOFs prevent Zn foil decomposition due to a highly concentrated electrolyte and 5 mA current density, the CoNi‐N‐C // CoNi‐BTC@Zn Zn‐air battery electrode exhibits 112 h of stable cycling (compared to Pt‐C + IrO_2_ // CoNi‐BTC@Zn) with reduced potential differences (Figure S21d). Additionally, Figure 6h shows how CoNi‐BTC MOFs are evenly deposited on ZnO surfaces throughout the charge–discharge process, increasing the longevity of ZABs. Examining the cycle durability at high capacities is essential for the practical use of pollutant‐free, earth‐abundant non‐noble metals, hybrid MOFs, and MOFs.

Using linear polarization experiments in the aqueous electrolyte, the effect of CoNi‐BTC MOFs coating on the corrosion behavior of Zn was examined. Electrochemical reactions can be used to explain corrosion occurrences. Corrosion rates, pitting patterns, and other crucial information can be obtained by carefully regulated current‐potential relation measurements. Compared to the traditional weight‐loss approach, the Tafel test is a faster experimental methodology and a popular polarization method for determining the corrosion rate [47, 48]. The typical plots of the applied voltage (V) versus the logarithm plots of the current density of corrosion (mA cm^−2^) are displayed in Figure 7a,b. On the first day, the corrosion potential for the bare and CoNi MOF‐plated zinc electrodes was −0.040096 and −0.03089 V, respectively. The corrosion current “Icorr” values for the bare zinc and CoNi‐BTC MOF‐plated zinc foils were determined to be 80.99 and 15.65 µ A on the first day, respectively. For the bare zinc and CoNi‐BTC MOF‐covered zinc electrodes, the ratios of Icorr and Ecorr are shown in Figure S30, respectively. It is commonly believed that a lower corrosion current also indicates a lesser rate of corrosion, while a larger positive corrosion potential indicates a decreased susceptibility to reactions caused by corrosion (such as evolution of H_2_ and the passivation driven by dissolved O_2_). In contrast to the Ni‐Co BTC MOF‐coated Zn foil sample, which showed very little of these changes, the bare Zn foil demonstrated considerable Zn corrosion and the creation of ZnO/Zn(OH)_2_, as seen by a rise in oxygen weight percentage and decrease in zinc weight percentage.

Before Zn‐air battery performance, we fabricated the CoNi‐MOF‐coated Zn anode through the drop‐casting method; it is a CoNi‐MOF@Zn anode. After trying an oven overnight at 60°C, we followed the hot‐pressing technique to fabricate a gas diffusion electrode (GDE), see in Figure 7c. This process enhances the interface between the CoNi‐MOF and the Zn plate, reducing contact resistance and improving specific capacity as well as long‐lasting Zn‐Air battery lifespan. For the following hot‐press have been check the thickness of the Zn‐air anode electrode of CoNi‐MOF@Zn through digital thickness gauge; it was 0.321 nm on without coating side and CoNi‐MOF coated side showing 0.421 nm (Figure 7d,e). This confirmed CoNi‐MOF@Zn anode electrode surface smoothness and controlled the thickness; therefore, CoNi‐MOF@Zn anode showed 1000 cycles with lower potential differences during charge–discharge stability and higher specific capacity values (Figure 6d,f). For further understanding, Figure 7f shows the without MOF coating Zn anode was dissolved much in 6 M KOH with 0.2 M zinc acetate, but after CoNi‐MOF coated Zn plate showed very little Zn surface dissolution. Also, Figure 7g shows all the information for the Zn anode coating process. Finally, after Zn‐air battery charge–discharge stability without a coated Zn anode and MOF‐coated CoNi‐MOF@Zn anode electrodes, as well as air cathode electrodes structural and chemical changes were analyzed by XRD, TEM, and XPS analyses.

### 3.5 Post‐Analysis of Corrosion

The AFM images in Figure S22a,b show the presence of Co‐Ni nanoparticles (CoNi‐N‐C air cathode) with different particle sizes because of the mixed hydroxide ions, while the specific discharge capacity in the Zn‐air battery electrolyte is present. Furthermore, the CoNi‐N‐C air cathode electrode displayed major planes of (111), (200), and (220) according to the XRD peaks shown in Figure S23a. Additionally, following Zn‐air battery stability, nickel oxyhydroxide planes for (104), (111), (006), (113), (114), and (214) with appropriate reference JCPDS card‐084‐1459, because hydroxide forms on metal surfaces during the stability of zinc‐air batteries. Additionally, Figure S23b shows the primary planes of CoNi‐MOF and Zn(OH)_2_ in the Zn foil and NiCo‐MOF@Zn anodes. This indicates that CoNi‐MOF is more stable because it controls the dissolution of Zn metal, which in some way raises the specific capacity values (Zn anode 724.9 mA h g_Zn_^−1^ and CoNi‐MOF@Zn 756.3 mA h g_Zn_^−1^).

Additionally, TEM images in Figures S24–S29 show cathode CoNi‐N‐C, anode CoNi‐BTC@Zn, and Zn foil electrodes after specific capacity and 1000‐cycles charge–discharge stability, respectively. Primarily, Figure S23 illustrates how the structural deformation of the cathode catalyst of CoNi‐N‐C electrodes was analyzed after ZABs specific capacity, which demonstrated lower catalyst leaching throughout the durability. Steady crystal planes (013), (102), and (006) for oxyhydroxide creation and particular EDS elemental mapping validate the TEM results, sustained CoNi generation with hybrid‐MOFs support (Figure S25). Recent research indicates that aqueous electrolytes with nearly neutral potential can prevent the production of carbonates and zinc dendrites. High ZnO concentrations produce excess zinc hydroxide after discharging and precipitation (Figure S26a–c), which makes the zinc electrode more resistant to passivation [49, 50]. Oxygen is released when zincate ions are converted to zinc, as demonstrated by crystal planes (102, 002‐ZnO) and EDS mapping of elements (Figure S26). The time‐dependent features of excess saturation by zinc ions in an alkaline solution are one of the biggest obstacles to the development of rechargeable ZABs.

The post‐morphology of CoNi‐BTC MOF coated on Zn anodes is depicted in Figure S27a–c, where the CoNi‐BTC@Zn electrode exhibits the CoNiO@C and ZnO nanocomposite with appropriate TEM crystal lattice spacings (111‐NiCo_2_O_4_; 002‐ZnO). Zn has a stronger mechanical interaction and less leaching with CoNi‐BTC composites, which increases the durability of ZABs, according to EDS elemental mapping (Figure S27d–g). Furthermore, we examined the cathode CoNi‐N‐C and anode CoNi‐BTC@Zn electrodes in Figures S28 and S29 after 1000 cycles of charge–discharge stability post‐morphology investigations. These images show that a high leaching (CoNi NPs) and broken‐down (Zn foil) metallic composites of cathode‐anode electrodes for charge–discharge stability after 1000 cycles when compared to specific capacity. More stable structures and composites confirmed the existence of elemental and all crystal lattice spacings for post‐charge–discharge stability, along with shorter durability despite the long‐term durability of both cathode CoNi‐N‐C and anode CoNi‐BTC@Zn catalysts.

**FIGURE 8:**
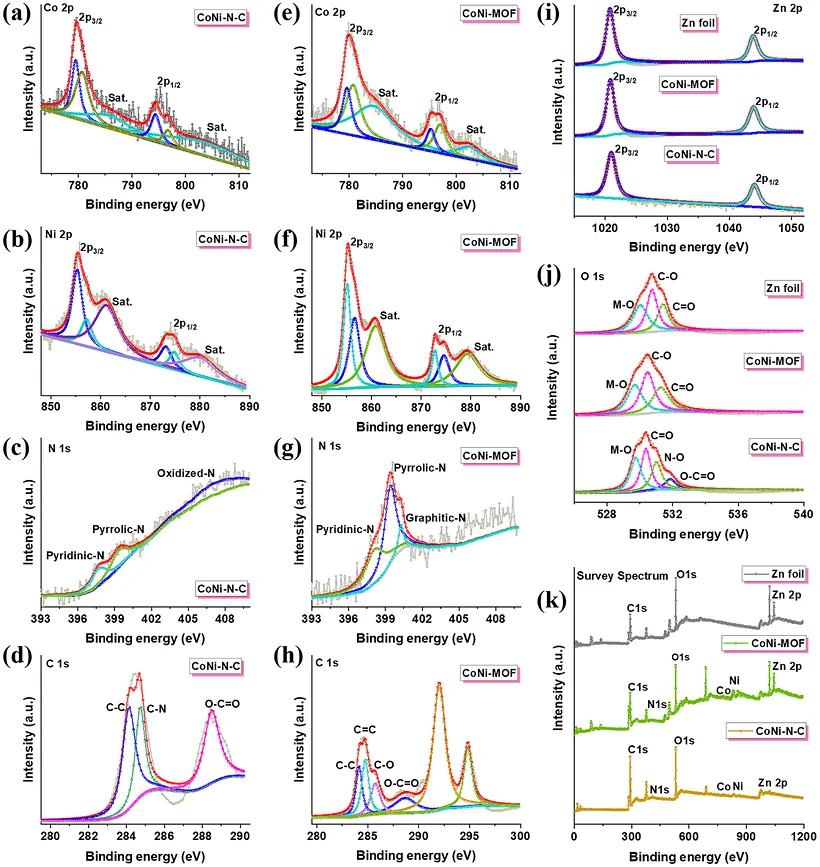
After 1000 cycles, Zn‐air battery high‐resolution XPS spectrum; (a–d) Co 2p, Ni 2p, N 1s, and C 1s of CoNi‐N‐C composite for cathode electrode. (e–h) Co 2p, Ni 2p, N 1s, and C 1s of CoNi‐MOF@Zn composite for anode electrode. (i) Zn 2p, (j) O 1s, and (k) survey spectrum of Zn foil, CoNi‐MOF@Zn, and CoNi‐N‐C electrodes.

Following 1000‐cycles charge–discharge stability tests, the sample was analyzed using XPS to look at modifications to surface species of the cathode CoNi‐N‐C, anode CoNi‐MOF@Zn, and anode Zn electrodes as activities declined. As shown in Figure 8k, XPS revealed notable alterations in the CoNi‐N‐C, anode CoNi‐MOF@Zn, and anode Zn in the survey spectrum. While the Co 2p, Ni 2p, Zn 2p, N 1s, O 1s, and C 1s peaks for cathode and anode electrodes changed considerably, the Co 2p, Ni 2p, N 1s, and C 1s peaks at the catalyst surface were almost the same (Figure 8a–d). Following Zn‐air battery charge–discharge stability, each of the peaks of pyridinic‐N and pyrrolic‐N was diminished, possibly enhancing Zn‐air battery performance. For anode CoNi‐MOF@Zn electrodes, Co and Ni atoms were more stable for further stabilization than cathode catalysts. The of CoNi‐MOF@Zn composites of the N 1s XPS peak spectra of CoNi‐MOF@Zn composites exhibit a discernible shift in the pyrrolic‐N peaks, as seen in Figure 8g. Additionally, the C 1s spectra are typically produced by the creation of additional hydroxides, as seen in Figure 8d,h.

Additionally, as shown in Figure 8i,j, the XPS spectra of Zn 2p showed a positive shift from 1044.0 eV to 1043.9 eV for cathode and anode electrodes. Understanding the chemistry of the zincate ion, Zn(OH)_4_^2−^(aq), in a solution that is alkaline is necessary to construct a secondary zinc electrode in a zinc‐air battery [49]. Negative XPS O 1s spectra for anode electrodes ranging from 530.4 eV (NiCo‐MOF@Zn) to 530.7 eV (Zn foil) revealed that the MOF coating on Zn anode somehow reduces the production of zincate ions. Lastly, this work strongly shows that the production of zincate ions is reduced, which makes us believe this confirmation will help future researchers improve their studies on Zn‐air batteries.

## 4 Conclusions

In conclusion, we have illustrated an efficient and profitable technique for regulating the structural composition of carbon‐supported electrocatalysts made from hybrid‐MOFs and MOFs as potential electrodes for Zn‐air batteries. In addition to increasing the necessary electron transit of Co‐Ni metals, the addition of hybrid‐MOFs and MOFs also served as a spatial barrier, increasing the spacing between atoms among Co‐Ni and preventing the leaching of CoNi NPs while encouraging the synthesis of CoNi‐N‐C and CoNi‐BTC MOFs. The modified CoNi‐N‐C catalyst produced a lower oxygen evolution overpotential of 1.48 V, an improved oxygen reduction half‐wave potential of 0.781 V, and a smaller potential difference of 0.699 V, according to electrochemical experiments. CoNi‐N‐C as cathode and CoNi‐BTC@Zn as anode produced a high specific capacity of 756.3 mA h g_Zn_^−1^, an exceptional peak power density of 290.6 mW cm^−2^, and great cycling durability over 1000 cycles in rechargeable zinc‐air batteries. According to post‐study XPS analysis, pyrrolic‐N reconstruction is one of the most sophisticated approaches for improving Zn‐air battery performance. This paper describes a low‐cost technique for creating CoNi nanoparticles in cathode‐anode electrodes using hybrid‐MOFs and MOFs carbon‐based electrocatalysts.

## Author Contributions

**Ramasamy Santhosh Kumar**; Conceptualization, experimental investigation, formal analysis, and Writing – review original draft. **Duraisamy Kaviyarasu** and **Dilmurod Sayfiddinov**; data visualization, formal analysis, and review of the original draft. **Murugavel Kathiresan** and **Arul Manuel Stephan**; review of original draft and data visualization. **Dong Jin Yoo**; Writing – review original draft, review of the original draft, acquisition of funding, and research supervision of work.

## Conflicts of Interest

The authors declare no conflict of interest.

## Supporting information




**Supporting File**: advs77650‐sup‐0001‐SuppMat.docx.

## Data Availability

The data that support the findings of this study are available from the corresponding author upon reasonable request.

## References

[1] Y. He , X. Yang , Y. Li , et al., “Atomically Dispersed Fe–Co Dual Metal Sites as Bifunctional Oxygen Electrocatalysts for Rechargeable and Flexible Zn–Air Batteries,” ACS Catalysis 12, no. 2 (2022): 1216–1227, 10.1021/acscatal.1c04550.

[2] Z. Song , J. Ding , B. Liu , et al., “A Rechargeable Zn–Air Battery With High Energy Efficiency and Long Life Enabled by a Highly Water‐Retentive Gel Electrolyte With Reaction Modifier,” Advanced Materials 32, no. 22 (2020): 1908127, 10.1002/adma.201908127. 32301217

[3] H. Li , L. Ma , C. Han , et al., “Advanced Rechargeable Zinc‐Based Batteries: Recent Progress and Future Perspectives,” Nano Energy 62 (2019): 550–587, 10.1016/j.nanoen.2019.05.059.

[4] J. Fu , Z. P. Cano , M. G. Park , A. Yu , M. Fowler , and Z. Chen , “Electrically Rechargeable Zinc–Air Batteries: Progress, Challenges, and Perspectives,” Advanced Materials 29, no. 7 (2017): 1604685, 10.1002/adma.201604685. 27892635

[5] D. Sayfiddinov , R. Santhosh Kumar , V. Sakthivel , et al., “Dual‐Functional PdAg Alloy Oxygen Electrocatalyst for Stable Operation in Zinc–Air Batteries and Proton Exchange Membrane Fuel Cells,” ACS Materials Letters 8, no. 1 (2026): 161–170, 10.1021/acsmaterialslett.5c01274.

[6] J. Wang , W. Liu , G. Luo , et al., “Synergistic Effect of Well‐Defined Dual Sites Boosting The Oxygen Reduction Reaction,” Energy & Environmental Science 11, no. 12 (2018): 3375–3379, 10.1039/C8EE02656D.

[7] H.‐F. Wang and Q. Xu , “Materials Design For Rechargeable Metal‐Air Batteries,” Matter 1 (2019): 565–595.

[8] W. Sun , F. Wang , B. Zhang , et al., “A Rechargeable Zinc‐Air Battery Based On Zinc Peroxide Chemistry,” Science 371, no. 6524 (2021): 46–51, 10.1126/science.abb9554. 33384369

[9] M. B. Poudel , M. P. Balanay , P. C. Lohani , K. Sekar , and D. J. Yoo , “Atomic Engineering of 3D Self‐Supported Bifunctional Oxygen Electrodes for Rechargeable Zinc‐Air Batteries and Fuel Cell Applications,” Advanced Energy Materials 14, no. 30 (2024): 2400347, 10.1002/aenm.202400347.

[10] M. Zhou , Y. Shi , H. Wu , et al., “Interfacial Electronic Tuning of Battery‐Recycling‐Derived Heterostructured Sulfides For Bifunctional Electrocatalysis in Zn‐Air Batteries,” Nano Energy 152 (2026): 111886, 10.1016/j.nanoen.2026.111886.

[11] D. E. Turney , J. W. Gallaway , G. G. Yadav , et al., “Rechargeable Zinc Alkaline Anodes for Long‐Cycle Energy Storage,” Chemistry of Materials 29, no. 11 (2017): 4819–4832, 10.1021/acs.chemmater.7b00754.

[12] F. Wang , O. Borodin , T. Gao , et al., “Highly Reversible Zinc Metal Anode For Aqueous Batteries,” Nature Materials 17, no. 6 (2018): 543–549, 10.1038/s41563-018-0063-z. 29662160

[13] A. R. Mainar , L. C. Colmenares , H.‐J. Grande , and J. A. Blázquez , “Enhancing the Cycle Life of a Zinc–Air Battery by Means of Electrolyte Additives and Zinc Surface Protection,” Batteries 4, no. 3 (2018): 46, 10.3390/batteries4030046.

[14] D. Ruan , Z. Cui , J. Fan , D. Wang , Y. Wu , and X. Ren , “Recent Advances in Electrolyte Molecular Design For Alkali Metal Batteries,” Chemical Science 15, no. 12 (2024): 4238–4274, 10.1039/D3SC06650A. 38516064 PMC10952095

[15] A. Iqbal , O. M. El‐Kadri , and N. M. Hamdan , “Insights Into Rechargeable Zn‐Air Batteries For Future Advancements in Energy Storing Technology,” Journal of Energy Storage 62 (2023): 106926, 10.1016/j.est.2023.106926.

[16] K. W. Leong , Y. Wang , M. Ni , W. Pan , S. Luo , and D. Y. C. Leung , “Rechargeable Zn‐Air Batteries: Recent Trends And Future Perspectives,” Renewable and Sustainable Energy Reviews 154 (2022): 111771, 10.1016/j.rser.2021.111771.

[17] Y. Zhou , H. Tong , Y. Wu , et al., “A Dendrite‐Free Zn Anode Co‐modified With in and ZnF_2_ for Long‐Life Zn‐Ion Capacitors,” ACS Applied Materials & Interfaces 14, no. 41 (2022): 46665–46672, 10.1021/acsami.2c13536. 36194838

[18] W. Guo , Z. Cong , Z. Guo , et al., “Dendrite‐Free Zn Anode With Dual Channel 3D Porous Frameworks For Rechargeable Zn Batteries,” Energy Storage Materials 30 (2020): 104–112, 10.1016/j.ensm.2020.04.038.

[19] J.‐H. Lee , R. Kim , S. Kim , et al., “Dendrite‐Free Zn Electrodeposition Triggered By Interatomic Orbital Hybridization of Zn And Single Vacancy Carbon Defects For Aqueous Zn‐Based Flow Batteries,” Energy & Environmental Science 13, no. 9 (2020): 2839–2848, 10.1039/D0EE00723D.

[20] H. Liu , J.‐G. Wang , W. Hua , et al., “Navigating Fast And Uniform Zinc Deposition Via A Versatile Metal–Organic Complex Interphase,” Energy & Environmental Science 15, no. 5 (2022): 1872–1881, 10.1039/D2EE00209D.

[21] H. Liu , J.‐G. Wang , W. Hua , et al., “Building Ohmic Contact Interfaces Toward Ultrastable Zn Metal Anodes,” Advanced Science 8 (2021): 2102612. 34672109 10.1002/advs.202102612 PMC8655195

[22] M. Gopalakrishnan , S. Ganesan , M. T. Nguyen , et al., “Critical Roles of Metal–Organic Frameworks in Improving The Zn Anode in Aqueous Zinc‐Ion Batteries,” Chemical Engineering Journal 457 (2023): 141334, 10.1016/j.cej.2023.141334.

[23] Y.‐S. Wei , M. Zhang , R. Zou , and Q. Xu , “Metal–Organic Framework‐Based Catalysts With Single Metal Sites,” Chemical Reviews 120, no. 21 (2020): 12089–12174, 10.1021/acs.chemrev.9b00757. 32356657

[24] D.‐D. Zhou , X.‐W. Zhang , Z.‐W. Mo , et al., “Adsorptive Separation of Carbon Dioxide: From Conventional Porous Materials To Metal–Organic Frameworks,” EnergyChem 1, no. 3 (2019): 100016, 10.1016/j.enchem.2019.100016.

[25] N. Li , Z. Chang , H. Huang , et al., “Specific K ^+^ Binding Sites as CO_2_ Traps in a Porous MOF for Enhanced CO_2_ Selective Sorption,” Small 15, no. 22 (2019): 1900426, 10.1002/smll.201900426. 30977961

[26] Y. Li , M. Cui , Z. Yin , S. Chen , and T. Ma , “Metal–Organic Framework Based Bifunctional Oxygen Electrocatalysts For Rechargeable Zinc–Air Batteries: Current Progress And Prospects,” Chemical Science 11, no. 43 (2020): 11646–11671, 10.1039/D0SC04684A. 34094409 PMC8163256

[27] H. Tan , Y. Zhou , S.‐Z. Qiao , and H. J. Fan , “Metal Organic Framework (MOF) in Aqueous Energy Devices,” Materials Today 48: 270–284, 10.1016/j.mattod.2021.03.011.

[28] J. Fu , R. Liang , G. Liu , et al., “Recent Progress in Electrically Rechargeable Zinc–Air Batteries,” Advanced Materials 31, no. 31 (2019): 1805230, 10.1002/adma.201805230. 30536643

[29] R. Santhosh Kumar , P. Mannu , V. Srinivasadesikan , N. Bhuvanendran , C.‐L. Dong , and D. J. Yoo , “Electrocatalyst for Enhanced Redox Reactions in Alkaline Zn–Air Batteries,” Small Science 6 (2026): 202500448. 10.1002/smsc.202500448 PMC12903980 41695890

[30] F. Cheng and J. Chen , “Metal–Air Batteries: From Oxygen Reduction Electrochemistry To Cathode Catalysts,” Chemical Society Reviews 41, no. 6 (2012): 2172–2192, 10.1039/c1cs15228a. 22254234

[31] J. F. Parker , C. N. Chervin , I. R. Pala , et al., “Rechargeable Nickel–3D Zinc Batteries: An Energy‐Dense, Safer Alternative To Lithium‐Ion,” Science 356, no. 6336 (2017): 415–418, 10.1126/science.aak9991. 28450638

[32] X. Wang , Y. Dai , P. Wang , and D. Yue , “Melamine Intercalation Approach For The Synthesis of C, N codoped net‐Like NiCo Oxides Composites And Its Application Towards Oxygen Evolution Reaction in Alkaline Solution,” International Journal of Hydrogen Energy 51 (2024): 1407–1416, 10.1016/j.ijhydene.2023.07.292.

[33] Z. Li , H. He , H. Cao , et al., “Atomic Co/Ni dual sites and Co/Ni alloy nanoparticles in N‐doped porous Janus‐Like carbon frameworks for bifunctional oxygen electrocatalysis,” Applied Catalysis B: Environmental 240 (2019): 112–121, 10.1016/j.apcatb.2018.08.074.

[34] S. Huang , W. Zhang , Q. Chen , et al., “N‐Doped Carbon Confined NiCo Alloy Hollow Spheres as an Efficient and Durable Oxygen Electrocatalyst for Zinc‐Air Batteries,” Chemistry—A European Journal 29, no. 30 (2023): 202300321, 10.1002/chem.202300321. 36890654

[35] V. Beermann , M. Gocyla , E. Willinger , et al., “Rh‐Doped Pt–Ni Octahedral Nanoparticles: Understanding the Correlation Between Elemental Distribution, Oxygen Reduction Reaction, and Shape Stability,” Nano Letters 16, no. 3 (2016): 1719–1725, 10.1021/acs.nanolett.5b04636. 26854940

[36] A. Muthurasu , R. Balaji , T. H. Ko , et al., “Atomically Dispersed Iron on Functionalized Boron Nitride Nanosheets for Efficient Oxygen Reduction in Proton Exchange Membrane Fuel Cells,” ACS Catalysis 15, no. 22 (2025): 18987–18994, 10.1021/acscatal.5c06792.

[37] C. Wang , H. Ren , Z. Wang , Q. Guan , Y. Liu , and W. Li , “A promising Single‐Atom Co‐N‐C Catalyst for Efficient CO_2_ Electroreduction And High‐Current Solar Conversion of CO_2_ to CO,” Applied Catalysis B: Environmental 304 (2022): 120958, 10.1016/j.apcatb.2021.120958.

[38] P. Li , W. Li , Y. Huang , Q. Huang , and S. Tian , “3D Hierarchical‐Architectured Nanoarray Electrode for Boosted and Sustained Urea Electro‐Oxidation,” Small 19, no. 30 (2023): 2300725, 10.1002/smll.202300725. 37035957

[39] L. Chen , Y. Zhu , D. Wang , J. Zhu , Y. Ren , and G. Chen , “Efficient Photocatalytic Degradation of RhB in Water via Co_3_O_4_ and F‐Doped Carbon Nitride Heterojunction,” ChemistrySelect 10 (2025): 202402822.

[40] H. Li , T. A. Ha , S. Jiang , et al., “N, F and S Doped Carbon Nanofibers Generated From Electrospun Polymerized Ionic Liquids For Metal‐Free Bifunctional Oxygen Electrocatalysis,” Electrochimica Acta 377 (2021): 138089, 10.1016/j.electacta.2021.138089.

[41] Q. Xue , Y. Wang , M. Jiang , et al., “Engineering Electronic Spin State of a CoNi Alloy for an Efficient Oxygen Reduction Reaction,” ACS Applied Energy Materials 6, no. 3 (2023): 1888–1896, 10.1021/acsaem.2c03831.

[42] P. Mannu , R. K. Dharman , T. T. T. Nga , et al., “Tuning of Oxygen Vacancies in Co_3_O_4_ Electrocatalyst for Effectiveness in Urea Oxidation and Water Splitting,” Small 21, no. 4 (2025): 2403744, 10.1002/smll.202403744. 39434462

[43] R. Santhosh Kumar , P. Thangavel , P. Mannu , et al., “Synergistic Metallic Co and Co‐N_4_ Active Sites in Surface‐Enriched Co‐NC Catalysts for Alkaline Zinc‐Air Batteries and Anion Exchange Membrane Water Electrolyzers,” Journal of Materials Chemistry A 14, no. 50 (2026): 34520–34532.

[44] I. Thaheem , S. Ali , M. Waqas , et al., “Electrochemical Performance of NiCo_2_O_4_ Spinel Cathodes For Intermediate Temperature Solid Oxide Fuel Cells,” physica status solidi 219 (2022): 2100542.

[45] R. Santhosh Kumar , S. C. Karthikeyan , S. Ramakrishnan , et al., “Anion Dependency of Spinel Type Cobalt Catalysts For Efficient Overall Water Splitting in An Acid Medium,” Chemical Engineering Journal 451 (2023): 138471, 10.1016/j.cej.2022.138471.

[46] Y. Zhang , J. Wang , M. Alfred , et al., “Recent Advances of Micro‐Nanofiber Materials For Rechargeable Zinc‐Air Batteries,” Energy Storage Materials 51 (2022): 181–211, 10.1016/j.ensm.2022.06.039.

[47] R. Huang , F. Wang , W. Han , et al., “Investigation of The Corrosion Behavior Influence of Carbon Interlayer On Aluminum Collector in Lithium‐Sulfur Battery System,” Electrochimica Acta 469 (2023): 143033, 10.1016/j.electacta.2023.143033.

[48] D. Sujatha , S. Karuppasamy , M. Krishnan , et al., “High‐Performing Zinc Air Batteries by Effective Mitigation of Zinc Anode Corrosion by MOF‐assisted Artificial Solid Electrolyte Interface,” EES Batteries (2026), 10.1039/d6eb00149a.

[49] R. Santhosh Kumar , S. Tamilarasi , A. M. Stephan , A. R. Kim , and D. J. Yoo , “CrS Doped MOF‐Derived Carbon Implanted CoNi Particles as Exceedingly Effectual Oxygen Electrocatalysts in Sustainable Zinc‐Air Batteries,” Small Methods 9, no. 4 (2025): 2401515, 10.1002/smtd.202401515. 39981777

[50] J.‐S. Lee , S. T. Kim , R. Cao , et al., “Metal–Air Batteries With High Energy Density: Li–Air Versus Zn–Air,” Advanced Energy Materials 1, no. 1 (2011): 34–50, 10.1002/aenm.201000010.

