# Supplementary material for: Hybrid Metal–Organic Framework‐Derived Dual Active Site Catalysts Enabling Surface‐Enriched Cathode and Modified Zinc Anode for Long‐Life Zinc–Air Batteries
Source: Adv Sci (Weinh). 2026 Sep 11:e77650. Online ahead of print. doi: 10.1002/advs.77650 (PMC13564279; doi:10.1002/advs.77650)
Supplement: Supplementary file 1 [file ADVS-9999-e77650-s001.docx]

**Supporting Information**

**Hybrid Metal**-**Organic Framework**-**Derived Dual Active Site Catalysts Enabling Surface-Enriched Cathode and Modified Zinc Anode for Long-Life Zinc–Air Batteries**

Ramasamy Santhosh Kumar^a^, Duraisami Kaviyarasu^a^, Dilmurod Sayfiddinov^a^, Murugavel Kathiresan^b^, Pandian Mannu^c^, Chung-Li Dong^c^, Arul Manuel Stephan^d^, Dong Jin Yoo^a,e*^

^a^Department of Energy Storage/Conversion Engineering of Graduate School, BK21 FOUR, Jeonbuk National University, Jeollabuk-do 54896, Republic of Korea

^b^P. C. Ray Department of Chemistry, National Institute of Technology Puducherry, Thiruvettakudy, Karaikal 609609, U. T. of Puducherry, India

^c^Research Center for X-ray Science, Department of Physics, Tamkang University, Tamsui, 25137 Taiwan

^d^CSIR- Central Electrochemical Research Institute, Karaikudi 630 003, India

^e^Department of Life Science, Hydrogen and Fuel Cell Research Center, Jeonbuk National University, Jeollabuk-do 54896, Republic of Korea

† *Corresponding Email ID: [djyoo@jbnu.ac.kr](mailto:djyoo@jbnu.ac.kr)

**Materials characterization**

Morphological characterization of all manufactured electrocatalysts was conducted using a highresolution transmission electron microscope and field emission scanning electron microscope with energy dispersive X-ray spectroscopy (EDS) (SUPRA 40 VP; Carl Zeiss, Germany) and HR-TEM (JEM-ARM200F, JEOL). X-ray diffraction (XRD) patterns of the produced electrocatalysts were evaluated using a PANalytical (X'PERT-PRO Powder) (model) and Cu K radiation (λ = 0.154 nm). The FT-IR spectra of CoNi/BTC-MOF were measured using a Perkin Elmer Frontier instrument with the standard KBr method. Inductively coupled plasma-optical emission spectrometry (ICP-OES) was conducted using a Thermo Fisher Scientific iCAP 7000 series to assess the loading of Zn and Co. The Raman spectrum of each manufactured electrocatalyst was obtained using elevated 3D mapping– scanning Raman spectroscopy with a NANO PHOTON (RAMAN Touch) outfitted with a 532-nm helium–neon laser at the Center for University-wide Research Facilities (CURF) of South Korea Jeonbuk National University (JBNU). The chemical states of the as-obtained materials were examined using X-ray photoelectron spectroscopy (XPS; Axis-Nova, Kratos Inc.). A Brunauer– Emmett–Teller (BET) Autosorb-iQ 2ST/MP physisorption analyzer was used to examine the surface area of the prepared catalysts at the South Korean KBSI (Korea Basic Science Institute of Jeonju Center).

**XAS analysis**

X-ray absorption spectroscopy (XAS) studies were performed at beamlines BL17C (Taiwan Light Source (TLS), National Synchrotron Radiation Research Center (NSRRC), Taiwan) and 12B2 (Spring 8, Japan). The XAS spectra were collected in the transmission mode. Raw data were analyzed following standard procedures, including pre-edge and post-edge background subtraction, normalization of the edge jump, and Fourier transformation. Soft XAS at the Fe and Co L-edges was performed at the 6-m high-energy spherical grating-monochromator HSGM beamline (BL20A at TLS) of the NSRRC. The spectra were recorded in the total electron yield mode using a microchannel plate detector.

**Electrochemical characterization**

**ORR measurements:** A Gamry Reference 600 Potentiostat/Galvanostat/ZRA equipped with a rotating ring–disk electrode rotator (RRDE-3A, ALS Co., Japan) was used to test the ORR activities of the prepared catalysts. The counter, reference, and working electrodes were a graphite rod, Ag/AgCl, and an RDE (5 mm: 0.19625 cm^2^), respectively. The reference electrode was standardized against the RHE for each experiment.

Approximately 3.0 mg of catalyst and 30 μL of 5% Nafion solution were dispersed in 1 mL of isopropyl alcohol and DI water (1:1) solution and sonicated for 60 min to generate a homogenous ink. A RDE (diameter of 5.0 mm) was drop-coated with approximately 20 μL of catalyst ink. For comparison, commercially available 20 wt.% Pt/C was purchased from Johnson-Matthey, UK, and the catalyst ink was prepared via a similar method and coated onto the RDE. CV (scan rate 50 mV s^-1^) and LSV (10 mV s^-1^) were performed with an N_2_- or O_2_-saturated 0.1 M KOH electrolyte. Additionally, LSV was conducted with varying RDE rotation speeds ranging from 200 to 2800 rpm with 0.2 to −0.8 V vs. Ag/AgCl. The electrolyte was bubbled with O_2_ for 30 min prior to each ORR test. To maintain O_2_ saturation, the O_2_ flow was maintained throughout the measurements. The methanol tolerance test was performed by chronoamperometry with a constant potential of −0.3 V vs. Ag/AgCl and 0.5 M methanol in O_2_-saturated 0.1 M KOH with constant rotation at 1600 rpm. A long-term cyclic durability test was performed using CV at a constant potential of 500 mV vs. Ag/AgCl at 1600 rpm in O_2_-saturated 0.1 M KOH for 10,000 cycles.

**Calculation of electron transfer during ORR**

Koutecky–Levich (K–L) plots were used to determine the number of electrons transferred at various potentials. (J^-1^ vs *ω*^−1/2^)

$$\frac{1}{J}=\frac{1}{J_{L}}+\frac{1}{J_{K}}=\frac{1}{B\omega^{1/2}}+\frac{1}{J_{K}} (S1)$$

$B=0.62 nF C_{0}D_{0}^{\frac{2}{3}}\nu^{-1/6}$	(S2)

where J, J_L_, and J_k_ are the measured, diffusion-limiting, and kinetic-limiting current densities, respectively.

**For OER measurements:**

The OER electrochemical performance was investigated using a three-electrode cell with a carbon paper-supported electrocatalyst as the working electrode. Ag/AgCl was used as the reference electrode, and a graphite rod was used as the counter electrode. The working electrode was first activated in 1.0 M KOH electrolyte for approximately 60 cycles until the curve sharpened. The OER polarization curves were documented at fixed scanning and sweep rates of 1 mV s^-1^ and obtained in 1.0 M KOH using linear sweep voltammetry (LSV). All the measured potentials were denoted based on the reversible hydrogen electrode (RHE) using the following equation:

E_RHE_ = E_AgCl_ + (0.197 + 0.059 × pH)	(S3)

Electrochemical impedance spectroscopy (EIS) was carried out within a frequency range of 100 kHz–0.1 Hz with an amplitude of 5 mV in 1.0 M KOH. Long-term durability tests were conducted using chronopotentiometric measurements. The anode and cathode sides of the water-splitting systems utilized identical electrocatalysts, and the working electrode for the OER was produced using the same fabrication approach.

**Capacitance measurements and comparison of ECSA**

Cyclic voltammetry (CV) was carried out to probe the electrochemical double layer capacitance (C_dl_) of various samples at non-Faradaic potentials between 0.05 and 0.25 V (vs. RHE in 1.0 M and 0.1 M KOH), with sweep rates of 10 to 100 mV s^-1^. The double-layer capacitance (C_dl_) values of the samples were estimated by plotting ΔJ = Ja - Jc against the CV scan rate, where the slope was twice C_dl_. The ECSA of each electrocatalyst was calculated as follows:

ECSA = C_dl_/Cs, (S4)

where Cs is the specific capacitance of the alkaline electrolyte (Cs = 0.04 mF cm^-2^). The roughness factor (RF) was calculated as follows:

RF = ECSA/GSA	(S5)

where GSA is the geometric surface area of the sample.

**Preparation of Ni-Co BTC MOF-coated Zn electrodes**

To prevent additional contamination, the high-quality zinc foil (Aldrich, USA) was first treated by washing with ethanol and drying at 60 °C. In a mortar and pestle, 0.80 g of Ni-Co MOF powder and 0.20 g of PVDF (Polyvinylidene fluoride) binder were combined with 10 mL of NMP (N-Methyl-2-pyrrolidone) to create the Ni-Co-BTC MOF slurry. Zn-foil was used to coat the produced slurry, which was then dried at 60 °C. To guaranty surface uniformity, the covered electrode was gently heat pressed. At room temperature, the contact angle of an aqueous electrolyte solution with uncoated zinc and Ni-Co-BTC MOF-coated zinc electrodes was measured using a Geninometer (USA).

**Assembly of 2032-type coin cells for corrosion studies**

For the surface analysis, zinc metal foil suitable for batteries was bought. After the zinc foils were formed into 14 mm diameter disks and carefully cleaned with acetone, 500 µL of the aqueous electrolyte (6.0 M KOH and 0.2 M zinc acetate) and a Celgard 2320 PP separator were used to assemble Zn||Zn coin cells. The OER investigations were conducted using the electrochemical analyzer (Auto Lab, Metrohm AG 2022-2025). For OER studies, a standard three-electrode setup was used: Zn foil/modified Zn foil as the working electrode, Pt foil as the counter electrode, and Hg/HgO as the reference electrode. The whole experimental analysis was conducted using DI water. The traditional coating procedure on Zn foil was used to prepare electrodes for the electrochemical OER investigations. Initially, the 1 x 1 cm^2^ Zn foil was ultrasonically cleaned for 15 minutes using Millipore water. The PVDF (polyvinylidene fluoride) adhesive was then used to create catalytic ink. After that, the covered sample was dried at 60 °C in a hot air oven. The OER performance of the dry and pristine electrodes was then assessed in 0.1M KOH.^[1]^

A Tafel test was used to examine the rate of corrosion and polarization resistance (RP) of the electrodes after the 2032-type Zn//Zn coin cells (Zn with both untreated and MOF-coated Zn) were put together with electrolytes and a Celgard 2320 PP separator. The Tafel test was conducted every 24 hours while the coin cells were kept at ambient temperature (25 °C). Potential scan rates of 1 mV s^-1^ were used for the polarization experiment. The corrosion current density (mA cm^-2^) logarithm plot and plot against applied potential (V) for the uncoated and plated zinc current collectors are shown in Figure 7.

Polarization tests were conducted at a scan rate of 5 mV/s at a specified potential range of 0 to 1 V (vs. Hg/HgO) in 0.1 M KOH using potentiostatic linear scan voltammetry (LSV). The Nernst equation was then used to translate any subsequent potential data with regard to RHE:

E(𝑣𝑠.𝑅𝐻𝐸) = E(𝑣𝑠.𝐻𝑔/𝐻𝑔𝑂)+0.059×𝑝𝐻+0.098𝑉	(S6)

**Zinc-Air Battery Test**

Carbon paper foam was coated (≈1 cm^2^) with Cur-Fe optimized catalyst ink (Toray Carbon Paper, TGP-H-60, 19 × 19 cm, Alfa Aesar). A zinc-air battery was constructed using an air cathode, zinc foil (0.25 mm thick, Alfa Aesar, UK), and an electrolyte of 6 M KOH and 0.2 M Zn (CH_3_CO_2_)_2_. The catalyst loading was kept at 1 mg cm^−2^. Durability tests for long-term chargedischarge cycles were examined using a Gamry 600 electrochemical workstation. For comparison, a similar process was utilized to create an air cathode electrode made of Pt/C (20 wt.%) and IrO_2_ (99.9%; Sigma-Aldrich) using a weight ratio of 1:1 for commercial Pt/C and IrO_2_. The power density (mW cm^-2^) and specific capacity (mA h g^-1^) of ZABs with Pt/C + IrO_2_ and Co-N-C-based air cathodes were determined by using Eqs S7 and S8.

$Power density \left( mW {cm}^{-2} \right)=Voltage \times Current density$	(S7)

$Specific capacity \left( \mathrm{mAh}g^{-1} \right)= \frac{Current \times Service hours}{Weight of consumed Zn}$	(S8)

**Figure S1.** (a, b) SEM-EDS images, (c, d, e) individual elemental mapping for Ni 2p, N 1s, and C 1s, (f) SEM-EDX spectrum of Ni-N-C catalyst.

**Figure S2.** (a, b) SEM-EDS images, (c, d, e) individual elemental mapping for Co 2p, N 1s, and C 1s, (f) SEM-EDX spectrum of Co-N-C catalyst.

**Figure S3.** (a) SEM-EDX spectrum of CoNi-N-C catalyst. (b) Particle size distribution corresponding AFM image of CoNi-N-C catalyst.

**Figure S4.** BET analysis of (a) N_2_ desorption and (b) pore diameter of Co-N-C and CoNi-N-C composites.

**Figure S5.** (a-d) TEM and HR-TEM analysis of CoNi-N-C catalyst (insert image; bar-chart of particle size distribution analyze through ImageJ software for corresponding TEM image).

**Figure S6.** (a) HR-TEM image, (b-c) lattice parameter of CoNi-N-C catalysts.

**Figure S7.** (a) XPS spectra of C 1s, (b) bar-chart of atomic percentages for Co-N-C, Ni-N-C, and CoNi-N-C composites of corresponding XPS results. (c) XAS studies of Co k-space spectra. (d) Crystal structure of Co-N-C catalyst.

**Figure S8.** ORR analysis cyclic voltammetry curves in 0.1 M KOH with 50 mV scan rate; (a) Pt-C, (b) Ni-N-C, (c) Co-N-C, and (d) CoNi-N-C electrocatalysts.

**Figure S9.** ORR analysis linear sweep voltammetry curves in 0.1 M KOH with 10 mV scan rate of 400 rpm to 2800 rpm speed; (a) Pt-C, (b) Ni-N-C, (c) Co-N-C, and (d) CoNi-N-C electrocatalysts.

**Figure S10.** (a-d) K-L plots of Pt-C, Ni-N-C, Co-N-C, and CoNi-N-C electrocatalysts. (e, f) LSV curve in a rotating ring-disk electrode (RRDE) setup of Co-N-C, Ni-N-C, and CoNi-N-C electrocatalysts.

**Figure S11.** (a) Chronoamperometry test: Methanol tolerance 0.1 M KOH with 1.0 M of methanol solution. (b) Linear sweep voltammetry curves for more than 10,000 cycles at a 500 mV scan rate before and after CV cyclic stability.

**Figure S12.** OER performance of cyclic voltammetry curves of prepared electrocatalysts in 0.1 M KOH at 50 mV scan rate; (a) IrO2, (b) Ni-N-C, (c) Co-N-C, and (d) CoNi-N-C electrocatalysts, respectively.

**Figure S13.** (a, b) OER performance of electrochemical impedance spectroscopy of IrO_2_, Ni-N-C, Co-N-C, and CoNi-N-C electrode.

**Figure S14.** Double-layer capacitance (C_dl_) measurements of CV curves of (a) IrO_2_, (b) Ni-N-C, (c) Co-N-C, and (d) CoNi-N-C electrocatalysts.

**Figure S15.** OER multi-step chronopotentiometry curve (insert image; before and after stability LSV curves) of CoNi-N-C electrocatalyst.

**Figure S16.** Bar-chart diagram of OER and ORR performances (a) overpotential and (b) half-wave potential values.

**Figure S17.** (a) Chemical structure of benzene -1,3,5-tricarboxylic acid, (b) FT-IR spectra of CoNi-BTC MOF composite.

**Figure S18.** (a, b) SEM images, (c-e) SEM-EDS elemental mapping and SEM-EDS spectra of CoNi-BTC MOF with presence of Co 2p, Ni 2p, O 1s, and C 1s elementals.

**Figure S19.** (a, b) Bright-field and dark-field TEM images, (c) TEM-EDS spectra with elemental percentages and overlap images of CoNi-BTC MOF composite.

**Figure S20.** (a) XPS survey spectra, (b, c) high-resolution XPS spectra of (b) C 1s, and (c) O 1s.

**Figure S21.** (a, b) Taking pictures of two Zn-air batteries connecting with red and green LED lights by using CoNi-N-C air cathode electrode and Zn anode electrodes, respectively. (c) Specific capacity of Pt-C + IrO_2_ // Zn and CoNi-N-C // Zn electrodes. (d) Charge-discharge cyclic stability of Pt-C+IrO_2_ // Zn@MOF and CoNi-N-C // Zn@MOF at 5 mA current density for 10 minutes charging and 10 minutes discharging.

**Figure S22.** After charge-discharge Zn-air battery stability AFM topographic results of CoNi-N-C electrode.

**Figure S23.** After specific capacity Zn-air battery stability XRD results of (a) CoNi-N-C air cathode electrode, (b) NiCo-MOF@Zn and Zn foil anode electrodes, after 1000 cycles charge-discharge stability air cathode CoNi-N-C and anode NiCo-MOF@Zn electrodes.

**Figure S24.** (a, b) After discharge Zn-air battery stability TEM results of CoNi-N-C electrocatalyst.

**Figure S25.** After discharge Zn-air battery stability TEM results of CoNi-N-C electrocatalyst; (a, b, c) TEM images, (d, e, f) HR-TEM and lattice parameters, (g) HAADF-TEM image, (h) TEM-EDS overlap image, (i) TEM-EDS elemental spectra, and (j) TEM-EDS mapping for following Co 2p, Ni 2p, N 1s, and C 1s elementals.

**Figure S26.** After discharge Zn-air battery stability TEM results of Zn anode electrode; (a-d) TEM, HR-TEM, SAED pattern, and lattice parameters images, (e) HAADF-TEM image, (f) TEM-EDS overlap image, and (g) TEM-EDS mapping for following Zn 2p, Co 2p, Ni 2p, N 1s, and O 1s elementals.

**Figure S27.** After discharge Zn-air battery stability TEM results of CoNi-BTC MOF@Zn anode electrode; (a-c) TEM, HR-TEM, SAED-FFT patterns, and lattice parameters images, (d) HAADF-TEM image, (e) TEM-EDS overlap image, (f) TEM-EDS elemental spectra, and (g) TEM-EDS mapping for following Zn 2p, Co 2p, Ni 2p, N 1s, and O 1s elementals.

**Figure S28.** TEM analysis for after 1000 cycles charge-discharge durability of CoNi-N-C air cathode electrodes; (a-e) TEM, HR-TEM, SAED pattern, and lattice parameter images, (f) TEM-EDS overlap image, TEM-EDS mapping for following Zn 2p, Co 2p, Ni 2p, N 1s, O 1s, and C 1s elementals, and TEM-EDS elemental spectra.

**Figure S29.** TEM analysis for after 1000 cycles charge-discharge durability of CoNi-N-C // Zn and Zn@CoNi-MOF anode electrodes; (a-f) TEM, HR-TEM, SAED pattern, and lattice parameter images, (g) HAADF-TEM image, (h) TEM-EDS overlap image, (i) TEM-EDS elemental spectra, and (j) TEM-EDS mapping for following Zn 2p, Co 2p, Ni 2p, N 1s, and O 1s elementals.

**Figure S30.** Tafel plots of (a) bare zinc (b) Ni-Co BTC MOF-coated zinc electrodes in aqueous

electrolyte as a function of time.

| Catalysts | ORR and OER study | | | Zn-Air battery performnces | | Charge-discharge cycles/time | **Ref.** |
| --- | --- | --- | --- | --- | --- | --- | --- |
|  | ORR  E_1/2_ (V) | OER  E_i=10_ (V) | ΔE | OCP/Power density  (V/mW cm^-2^) | Spec. capacity  (mA h g_zn_^-1^)  10 mA |  |  |
| CoNi-NC | 0.781 | 1.48 | 0.699 | 1.45/290.6 | 724.9 Zn  756.3 Zn-MOF | 1000@  333.3h | **This work** |
| Co-NC-800 | 0.93 | - | - | 1.44/109.5 | 657.2@20 mA | 200@33 | ^[2]^ |
| Co-N-C@GNP | 0.86 | 1.65 | 0.79 | 1.60/236.2 | - | 94@5 mA | ^[3]^ |
| Fe-Co_4_N@N-C | 0.83 | 1.62 | 0.79 | 1.46/105 | 806@5 mA | 220/36h | ^[4]^ |
| Ni_3_Fe/Co-N-C | 0.79 | 1.54 | 0.75 | 1.39/68 | - | 100/65h | ^[5]^ |
| Co/ZnCo_2_O_4_@NC | 0.90 | 1.60 | 0.7 | 1.47/305 | 922@20 mA | 103h | ^[6]^ |
| FeCo–NC | 0.877 | 1.579 | 0.702 | -/372 | - | 190h | ^[7]^ |
| Co/Ni-NC | 0.895 | 1.591 | 0.696 | 1.55/155.9 | 771@10 mA | 340/340h | ^[8]^ |
| Fe/Fe-N-C | 0.881 | 1.654 | 0.773 | 1.451/181 | 741@10 mA | 210/210h | ^[9]^ |
| Ni_3_Fe-NCNTs-800 | 0.862 | 1.583 | 0.721 | 1.51/211 | 806@10 mA | 1350/225h | ^[10]^ |
| FeNi_3_@NC | 0.783 | 1.522 | 0.739 | 1.48/149.7 | 658@10 mA | 1680/280h | ^[11]^ |
| FeNi-NC@CNT | 0.90 | 1.60 | 0.69 | 1.44/218 | 782@15 mA | 600/300h | ^[12]^ |
| FeNi_3_@NC | 0.86 | 1.507 | 0.647 | 1.39/139 | 756@10 mA | 30/30h | ^[13]^ |
| Fe/Ni-NC@PDA | 0.89 | 1.56 | 0.67 | 1.56/140 | 770@10 mA | 156h | ^[14]^ |
| NiCo@N−C HS | 0.77 | 1.54 | 0.77 | 1.35/100 | 788.7@10 mA | 200h | ^[15]^ |
| NiCo/MNC | 0.83 | 1.61 | 0.78 | 1.42/135.2 | 701.7@ 20 mA | 115h | ^[16]^ |
| NiCo–NG | 0.793 | 1.536 | 0.743 | 1.403/181.9 | 804.2@25 mA | 3000/500h | ^[17]^ |

**Table S1.** Electrochemical OER and ORR performances with alkaline Zn-Air battery for using M-N-C electrocatalysts in 0.1 M KOH and 0.2 M zinc-acetate containing 6 M KOH solutions.

**References**

[1]	D. Sujatha, S. Karuppasamy, M. Krishnan, K. Pranav, S. K. Panda, M. Kathiresan, S. K. Ramasamy, A. L. Natarajan, D. J. Yoo, A. M. Stephan, *EES Batteries* **2026**.

[2]	S. Zhao, L. Ban, J. Zhang, W. Yi, W. Sun, Z. Zhu, *Chemical Engineering Journal* **2021**, *409*, 128171.

[3]	Y. Liu, Z. Li, S. Wang, J. Xuan, D. Xiong, L. Zhou, J. Zhou, J. Wang, Y. Yang, Y. Du, *Nano Research* **2023**, *16*, 8893-8901.

[4]	Q. Xu, H. Jiang, Y. Li, D. Liang, Y. Hu, C. Li, *Applied Catalysis B: Environmental* **2019**, *256*, 117893.

[5]	J. Tan, T. Thomas, J. Liu, L. Yang, L. Pan, R. Cao, H. Shen, J. Wang, J. Liu, M. Yang, *Chemical Engineering Journal* **2020**, *395*, 125151.

[6]	L. Yan, Z. Xu, W. Hu, J. Ning, Y. Zhong, Y. Hu, *Nano Energy* **2021**, *82*, 105710.

[7]	Y. He, X. Yang, Y. Li, L. Liu, S. Guo, C. Shu, F. Liu, Y. Liu, Q. Tan, G. Wu, *ACS Catalysis* **2022**, *12*, 1216-1227.

[8]	Y. Yang, B. Li, Y. Liang, W. Ni, X. Li, G. Shen, L. Xu, Z. Chen, C. Zhu, J.-X. Liang, S. Zhang, *Advanced Science* **2024**, *11*, 2310231.

[9]	X. Zheng, X. Cao, Z. Sun, K. Zeng, J. Yan, P. Strasser, X. Chen, S. Sun, R. Yang, *Applied Catalysis B: Environmental* **2020**, *272*, 118967.

[10]	Q. Yan, X. Duan, Y. Liu, F. Ge, H. Zheng, *Journal of Materials Chemistry A* **2023**, *11*, 1430-1438.

[11]	X. Xie, H. Peng, K. Sun, X. Lei, R. Zhu, Z. Zhang, G. Ma, Z. Lei, *Chemical Engineering Journal* **2023**, *452*, 139253.

[12]	Z. Chen, W. Cheng, K. Cao, M. Jin, S. Rahali, S. A. Chala, E. Ebrahimi, N. Ma, R. Liu, K. Lakshmanan, C. Y. Chang, C. C. Cheung, H. Luo, Y. Wang, B. J. Hwang, C. Streb, *Small (Weinheim an der Bergstrasse, Germany)* **2025**, *21*, e2409161.

[13]	D. Chen, J. Zhu, X. Mu, R. Cheng, W. Li, S. Liu, Z. Pu, C. Lin, S. Mu, *Applied Catalysis B: Environment and Energy* **2020**, *268*, 118729.

[14]	S. Tian, L. Chen, S. Wu, J. Li, J. Yang, H. Zhang, L. Wang, Y. Sun, L. Wang, X. Meng, Q. Yang, *Chemical Engineering Journal* **2025**, *520*, 166120.

[15]	S. Huang, W. Zhang, Q. Chen, S. Zhou, L. Sun, L. Sha, G. Zhuang, P. Wang, X. Han, *Chemistry – A European Journal* **2023**, *29*, e202300321.

[16]	D. Deng, Y. Tian, H. Li, L. Xu, J. Qian, J. Pang, B. Wang, Q. Zhang, H. Li, *Journal of Alloys and Compounds* **2019**, *797*, 1041-1049.

[17]	B. Zhonghua, J. Li, J. Zhang, Q. Zhang, J. Li, F. Yang, S. Luo, *Microporous and Mesoporous Materials* **2024**, *380*, 113292.
